# Reliable Criterion Retrieval for Sensor-Instrumented Road Infrastructure: Diagnosing and Correcting a Title-Framing Bias in Dense Retrieval over Korean Design Documents

**DOI:** 10.3390/s26175683

**Published:** 2026-09-07

**Authors:** Byeong-Cheol Kim, Byung-Jik Son

**Affiliations:** 1 Department of Structural Engineering, Korea Institute of Civil Engineering and Building Technology (KICT), Goyang 10223, Republic of Korea; 2 Department of Disaster Safety & Fire, Konyang University, Nonsan 32992, Republic of Korea

**Keywords:** retrieval-augmented generation, dense retrieval, framing bias, hybrid retrieval, HWPX parsing, structural health monitoring, intelligent sensing, infrastructure inspection, design standards

## Abstract

Retrieval-augmented generation increasingly serves as the knowledge backbone for sensor-informed infrastructure decisions, where a field engineer must retrieve the clause stating a design criterion, not a document about the topic. In twenty years (2005–2024) of Korea Expressway Corporation design-practice guidelines (HWP/HWPX), we identify a framing bias in dense retrieval as follows: embedding models over-weight the topical aboutness of a section title relative to the criterion in its body. The corpus invites this failure as follows: 63.2% of sections carry plan-framed titles, yet 86.4% of them carry criterion-type language in the body. A controlled counterfactual (*n* = 80) holding the body fixed and rewriting only the title isolates the effect (Δcos = 0.030, *d_z_* = 1.63, *p* = 1.1 × 10^−14^), reproduces it on a second embedding family, and decomposes it into term-frequency, early-position, and title-framing components; the framing residual (*d_z_* = 0.53) survives primacy controls. A length- and frequency-matched neutral-token control splits that residual further into a token-composition component that replicates on both embedding families and a plan-framing component that reaches significance on one (*d_z_* = 0.46). The bias buries framing-prone criterion documents by tens to hundreds of ranks; standard remedies are partial. We propose Criterion-Aware Retrieval (CAR), which hypothesizes the sought criterion at query time; on the 21 low-overlap queries that the bias hits hardest it outperforms both BM25 and the weighted-RRF hybrid after Holm correction (MRR 0.271 vs. 0.048 and 0.120). A cross-encoder reranker ranks better still (0.376) at no language-model cost but cannot exceed the recall of the pool it reorders (0.714 against CAR’s 0.857): the two address different failure modes, and widening the pool is what the framing bias calls for. A parsing-pathway comparison shows that the structured pathways measured are near-lossless while PDF loses table content, justifying our HWPX-derived ground truth.

## 1. Introduction

Retrieval-augmented generation (RAG) increasingly underpins engineering decision-making. Its reliability hinges on the following easily overlooked step: whether semantic retrieval actually returns the passage that *contains* the answer. We study this in a demanding real-world setting as follows: the design-practice guidelines of the Korea Expressway Corporation, a twenty-year corpus (2005–2024) of Korean government technical documents in the HWP/HWPX format. These documents codify the criteria that govern how highways and bridges are built, inspected, and maintained, and in an era of infrastructure digital twins and sensor-instrumented inspection they act as the authoritative reference layer. When a field engineer asks how densely to place inspection sensors on a fixed bridge gantry, or a digital-twin module needs the governing installation standard for a component, the retrieval system must return the clause that *states* the criterion, not a document that is merely *about* the topic.

**Figure 1:**
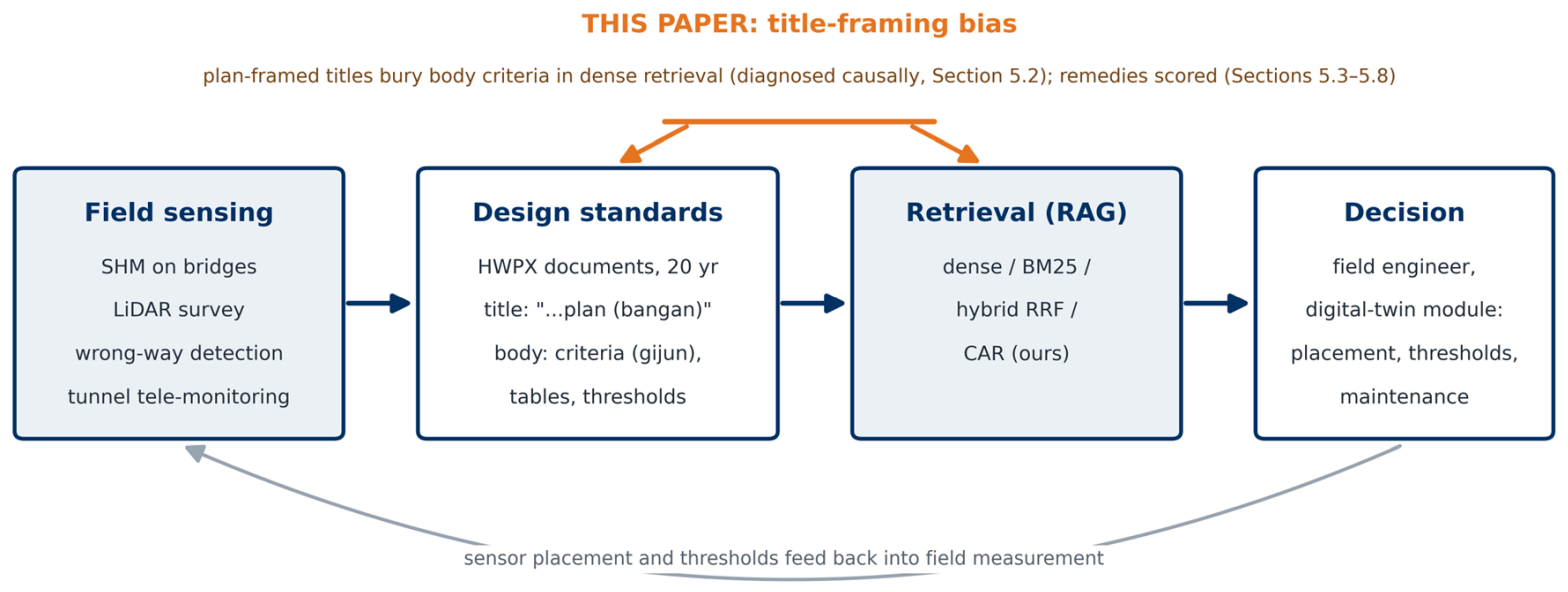
The standards-retrieval layer in sensor-instrumented road infrastructure. Field sensing both produces and consumes parameters that are codified as binding criteria in HWPX design-practice documents; engineers and digital-twin modules reach those criteria through retrieval. This paper diagnoses a title-framing bias at the document-retrieval link (plan-framed titles bury body criteria), scores the standard remedies, and adds a targeted query-time correction (CAR).

These specifications encode parameters that sensing systems both produce and depend on: design speeds from vehicle-detection surveys, installation criteria for radar and loop wrong-way detectors, LiDAR survey-accuracy standards, the placement of the fixed inspection facilities that carry structural-health sensors, and discharge thresholds for tunnel water-quality tele-monitoring. The retrieval step is the last link between a field measurement and the decision it informs. An error here does not stay an abstract search miss; it propagates into sensor-placement and maintenance decisions on physical infrastructure. Retrieval-augmented pipelines are accordingly becoming standard interfaces to sensing and operational data as follows: for maritime AIoT fleets [1], industrial assembly diagnosis [2], and query over building data [3]. The sensing side of this loop is itself advancing quickly, including the physical layer: structured-light sources such as vortex-beam and breathing-soliton oscillators are reaching sub-100-fs regimes with potential application in high-resolution optical sensing and metrology [4,5]. We cite these as an indication of how fast the instrument layer is moving, not as method for the present work, which concerns document retrieval rather than optical-source design. This work addresses the standards-document layer that such sensor-facing systems ultimately consult (Figure 1) and the specific way its retrieval can fail.

A word on scope. What we evaluate is document retrieval over a standards corpus; we do not evaluate a sensing system, and no measurement in this paper is taken from an instrument. The sensor connection is one of dependency: these documents fix the parameters that sensing and monitoring systems are specified and operated against, so a retrieval failure here propagates into decisions made about instrumentation. We test that this dependency is not merely rhetorical by isolating the 26 questions whose criterion a sensing system produces or consumes and reporting retrieval on them (Section 5.10), but that stratum is small and we report it descriptively throughout.

A RAG pipeline passes each document through parsing, chunking, embedding, and retrieval. Faithful parsing matters, but for this document class we find the dominant factor is subtler: how indexing encodes a section’s structure, and specifically the weight that dense retrieval places on the title relative to the body. We therefore focus on this structural effect rather than a surface-format comparison across export pathways (HML, HTML, and PDF), which we leave to future work; HWPX parsing is used here only to obtain reliable ground truth.

Dense retrieval fails on this corpus in a specific and consequential way. Korean administrative documents are dominated by *plan-framed* titles such as *bangan* (방안, “plan”) and *gaeseon* (개선, “improvement”), while the binding numerical criteria sit in the body under terms such as *gijun* (기준, “standard”). Across 1388 unique document sections, 877 (63.2%) carry plan-type titles against only 135 (9.7%) with standard-type titles, and the token *bangan* appears 614 times versus 358 for *gijun*. Yet 86.4% of plan-titled sections do contain criterion-type language in their body. The mismatch between title framing and body content is the rule, not the exception.

A motivating case makes the failure concrete. A section titled “교량 고정식 점검시설 확충방안” (an expansion *plan* for fixed bridge inspection facilities) gives the actual installation standards only in its body. A query for the “설치기준” (installation standard) of these facilities should retrieve this section, but dense retrieval pushes it down—over the full operational index, the section surfaces only at rank 78 of the ~1500 candidates returned for this query—because the embedding is drawn to the plan-framed title rather than to the criterion the user needs. The system returns what the document is *about* over what it *contains*, an aboutness-versus-containment mismatch that recurs throughout the corpus.

To show this is a causal effect of title framing rather than a vocabulary artifact, we ran a controlled counterfactual probe (*n* = 80, sampled across 2005–2024 from 566 eligible cases). For each section we constructed three variants alongside the original—rewriting only the title into criterion framing or keeping the plan title and adding one frequency-matched criterion mention at the body’s end or as its first line—and measured each variant’s cosine similarity to a criterion query. Moving the criterion term into the title raised similarity by Δ = 0.0303 in 78 of 80 cases (Wilcoxon *p* = 1.1 × 10^−14^, *d_z_* = 1.63). Frequency- and position-matched controls decompose this shift as follows: raw term frequency contributes +0.013, early body position +0.009, and the title framing itself a further +0.008 (74% of cases, *p* = 2.2 × 10^−5^, and *d_z_* = 0.53), a residual that survives controls for the primacy bias recently documented for dense encoders [6,7]. Two further controls decompose that residual rather than attribute it to a single cause: it is not explained by title position, and it divides into a token-composition part and a plan-framing part whose cross-model behavior differs (Section 5.2). The effect reproduced on an independent embedding family (Cohere multilingual-v3.0: Δ = 0.0206, 98%, *p* = 9.3 × 10^−15^, and *d_z_* = 1.64), ruling out a single-model artifact.

This work makes the following three contributions:

**C1 (Insight).** We identify and causally isolate a *framing bias* in dense retrieval over Korean administrative documents as follows: embeddings over-weight title aboutness relative to the criterial containment in body text. We separate this from frequency-based vocabulary mismatch through controlled counterfactuals and reproduce it across two embedding families. The finding reproduces classical *aboutness* theory [8] in neural retrieval and Korean technical documents and stands in directional contrast to the literal-bias collapse reported for dense retrievers [9].

**C2 (Prescription).** We examine corrections of differing maturity. At *retrieval time*, a weighted-RRF hybrid with a Korean morphological tokenizer improves significantly over dense alone, though on lexical cell-value QA it does not surpass BM25. At *index time*, **Criterial Reframing** surfaces a section’s body criterion in its indexed header as follows: under an oracle condition it cuts the target’s mean rank from 254.8 to 4.6 and worsens no case, establishing the mechanism’s ceiling. Yet no query-independent variant transfers to end-to-end QA (n.s.): the index cannot know which criterion a query will seek. We close that gap at *query time* with **Criterion-Aware Retrieval** (CAR, Section 5.8). Unlike generic expansion such as doc2query [10] and HyDE [11], CAR hypothesizes the specific criterion a query seeks; in the low-overlap regime the bias creates, it outperforms both BM25 and the hybrid after multiple-comparison correction. We also measure the strongest standard alternative rather than arguing against it from theory: a cross-encoder reranker achieves better ranking than CAR at no language-model cost but is bounded by the recall of the pool it reorders, which CAR raises. The contribution is therefore a complement to reranking, not a substitute for it.

**C3 (Engineering).** We build cell-coordinate ground truth for this document class and use it to quantify what one widely used ingestion pathway loses. An HWPX structure-aware parser recovers that ground truth directly from the source XML (26,757 tables) and against it we score *table preservation*. This is not yet the four-format comparison the question ultimately calls for: HWPX and PDF are measured corpus-wide, the HML arm rests on the single export currently available, and the HTML pathway is not measured at all (Section 6.5). Structured formats are near-lossless: HWPX, the source, defines the 100% reference, and an HML parser recovers 97.3% of large-table (≥2 × 2) cell content on a single-document probe. PDF, the format many pipelines default to, recovers 91.7% of large-table cell content but detects under half of all tables, and its recovery varies from 75% to 98% across the eight chapters. This justifies the HWPX-derived ground truth and shows that the PDF pathway, specifically, drops a measurable share of tabular criterion content—a claim about one pathway and one extractor pair, not a ranking of all four formats (corpus-scale HML and the HTML pathway require Hangul re-export; alternative extractors such as Camelot or Docling are likewise unmeasured; both are left to future work).

Korea’s ongoing government policy shift from the legacy HWP binary format to the XML-based HWPX makes principled parsing and retrieval over this document class an immediate practical concern.

The remainder of this paper is organized as follows. Section 2 reviews related work on dense retrieval, retrieval bias, and aboutness theory. Section 3 presents our problem framework and the counterfactual probe design. Section 4 describes the corpus, parsing, and experimental setup. Section 5 reports the following results: the framing-bias probe, the retrieval comparisons, the reframing and prevalence studies, parsing fidelity, and Criterion-Aware Retrieval. Section 6 discusses implications for sensor-driven infrastructure systems and the limitations of our study, and Section 7 concludes.

## 2. Related Work

Our work connects the following five strands of prior research: biases in dense neural retrieval, the information-science theory of aboutness, the vocabulary-mismatch problem, hybrid retrieval and Korean tokenization, and document-parsing benchmarks. We position our framing-bias finding against each.

### 2.1. Biases in Dense Retrieval

Dense retrievers built on bi-encoder architectures, such as DPR [12], embed queries and passages into a shared vector space and retrieve by cosine or inner-product similarity. While effective on open-domain benchmarks, their behavior on heterogeneous collections is uneven: BEIR [13] showed that dense models frequently underperform BM25 under zero-shot transfer, especially on domains with specialized terminology.

Recent work has begun to characterize *why*. Fayyaz et al. [9], in “Collapse of Dense Retrievers,” document systematic biases toward short, early-positioned, and literal lexical matches, where dense encoders over-weight surface tokens shared between query and passage. Our findings are directionally complementary, and in one respect opposite. Where literal bias over-weights surface lexical overlap, the framing bias we isolate lets the abstract title of an administrative section outweigh its concrete body criteria: a query for a design criterion is pulled toward a section whose heading frames it as a “plan” (방안), and even when the criterion (기준) is literally present in the body. The two phenomena are not contradictory. Both reflect dense encoders keying on salient, early, or framing-level signal rather than the full evidential content, but they pull in different directions on the lexical axis, which we make precise with a controlled counterfactual rather than an observational correlation. A rapidly growing companion line documents *positional* bias as follows: dense encoders over-weight early document content (“dwell in the beginning”) [6], a primacy effect confirmed across ten embedding model families [7] and traced in part to the positional distribution of training data [14]. Because a title is also simply the *earliest* text in a document, any title effect risks reduction to this known primacy bias. Our counterfactual design (Section 3.4) is built to separate the two and finds a title-framing effect that persists beyond early position and traces to the framing content rather than the title slot as such.

### 2.2. Aboutness, Topicality, and Relevance

The phenomenon we observe is best understood through the information-science distinction between what a document is *about* and what it *contains*. Hjørland [8], in his theory of aboutness, topicality, and relevance, argues that aboutness is a constructed, subject-level judgment that need not coincide with the propositions a document actually states. Titles are a canonical aboutness signal. Our framing bias is, in effect, a neural-retrieval instantiation of an aboutness–containment mismatch: the embedding model encodes a section’s heading-level aboutness (“this section is about a plan”) and under-weights criterial containment (“this section states the following numerical standard”). Anchoring the finding in this established theory keeps us from over-claiming novelty. We do not posit a wholly new bias; we show that a long-recognized aboutness/topicality gap is reproduced by modern neural encoders, specifically in Korean government technical documents, with a measurable and causally isolated effect size.

### 2.3. The Vocabulary Problem

One might object that our effect merely restates the classic vocabulary-mismatch problem [15], in which users and documents name the same concept with different words. It does not: in the vocabulary problem the sought term is *absent* from the relevant document, whereas here the criterion term (기준) is present in the body of 86.4% of the plan-titled sections that make up our population. Failure is one of weighting, not of lexical absence. Section 3.4 sets out the control design that establishes this.

### 2.4. Hybrid Retrieval and Korean Tokenization

A standard remedy for dense-retriever weaknesses is to combine lexical and semantic signals. Reciprocal Rank Fusion [16] merges ranked lists without score calibration and is robust across retrievers; learned sparse models such as SPLADE [17] expand queries and documents into weighted lexical terms, and cross-encoder reranking [18] refines top-k candidates. Recent work tunes the lexical–semantic balance dynamically as follows: DAT [19] adjusts the fusion weight α per query. Our prescriptive contribution adopts weighted RRF (k = 60) over BM25 and dense lists with a tunable α and reports both the gain over dense alone and the negative result that the hybrid does not beat BM25 on our lexically keyed QA set. For the lexical channel, Korean’s agglutinative morphology makes whitespace tokenization inadequate; prior work on Korean tokenization for neural and IR models [20] motivates morpheme-aware processing. We use a Kiwi morphological analyzer combined with domain-specific normalization for BM25 (BM25Okapi, k1 = 1.5, and b = 0.75), a choice tailored to the orthography and morphology of Korean administrative text.

### 2.5. Document Parsing and RAG on Office Formats

End-to-end RAG quality is bounded by upstream parsing fidelity. Document-parsing benchmarks such as OmniDocBench [21] systematically evaluate layout, table, and formula extraction, but for PDF and mainstream office formats. The Korean HWP/HWPX family, the de facto standard for government documents, remains a blank spot: no comparable benchmark characterizes parsing fidelity across HWPX, HML, HTML, and PDF export paths. We begin to fill this gap by using the HWPX parser to extract cell-coordinate ground truth directly from the source XML (26,757 tables across the corpus), which enables ground-truth-based evaluation; a full four-format comparison is left to future work. Finally, RAG over technical and sensor-adjacent documentation is an active line within sensing venues themselves: recent work in this journal spans natural-language access to maritime AIoT operational data [1], graph-enhanced retrieval for industrial assembly diagnosis [2], retrieval-augmented query over building data [3], and—closest to our setting—emergency-operation-scheme generation for urban-rail door systems that retrieves from operating regulations and procedures [22]. That line interfaces with *measured or machine data* or consumes document retrieval as a pipeline component; whether the retrieval layer itself reliably surfaces the *governing criterion* from the authoritative standards documents that sensor-instrumented infrastructure is designed, inspected, and maintained against has not, to our knowledge, been examined. Parsing-fidelity-aware and bias-aware retrieval over Korean infrastructure design standards is the gap this work fills.

## 3. Problem Framework: Aboutness vs. Criterial Containment

### 3.1. Two Senses of “What a Document Is About”

We distinguish two ways a technical document relates to an information need. The first is *aboutness*: the topic a document announces about itself, conventionally signaled by its title [8]. The second is *criterial containment*: whether the document body actually contains the specific decision criterion a reader seeks, such as a numeric threshold, a tolerance, or a spacing rule. In engineering practice these come apart routinely. A section announced as a “plan to expand bridge inspection facilities” (aboutness) may nonetheless be the authoritative source of the *installation criteria* an inspector needs (criterial containment). The retrieval target is containment; the dominant surface signal is aboutness.

### 3.2. Korean Administrative Title Conventions

Korean government technical documents follow strong section-naming conventions that sharpen this gap. Titles are predominantly framed as *plans* or *improvements* (방안, 개선) rather than as *standards* or *guidelines* (기준, 지침). In our corpus of 1388 unique document sections, plan-type titles dominate at 877 (63.2%), while standard-type titles account for only 135 (9.7%); 292 (21.0%) are mixed and 84 (6.1%) carry neither marker. The lexical frequencies are stark: *bangan* (방안, “plan,” 614) and *gaeseon* (개선, “improvement,” 516) far outnumber *gijun* (기준, “criterion/standard,” 358). The criterion is not absent from these documents; it is simply not in the title. Fully 86.4% of plan-type sections contain *gijun*-type criterial language in their body. The naming convention thus systematically separates aboutness (plan framing in the title) from criterial containment (standards in the body), defining a large population (566 control-eligible cases) in which the two signals conflict.

### 3.3. The Framing-Bias Hypothesis

We hypothesize that dense embedding models over-weight the title’s aboutness signal relative to the body’s criterial containment. When a user queries for a *criterion* (*topic* + 기준), a document whose title is plan-framed is pushed down in similarity space even though its body contains the sought criterion, while a title-framed match is pulled up. Dense retrieval, in other words, partially answers “what is this section announced to be about?” when the user asked “which section contains this criterion?”.

We cast this as a falsifiable, counterfactual proposition rather than an observational correlation:

> **P1 (Title-framing counterfactual).** Holding the document body fixed and altering only the title framing changes a dense retriever’s similarity to a criterion query.

Concretely, for a fixed body we construct two title variants, an original plan-framed title (orig) and a criterion-framed title (cf), and measure cos(*query*, *variant*). If aboutness is over-weighted, then cos(*query*, cf) > cos(*query*, orig) even though the body, and hence the criterial containment, is identical across variants. This isolates a *causal* effect of title framing, which an observational rank study over the live corpus cannot establish.

### 3.4. A Residual Distinct from Vocabulary Mismatch

The immediate objection is that P1 merely restates the classic *vocabulary mismatch* problem [15]: the query term 기준 is simply present in cf and absent in orig, so higher similarity is unremarkable. We therefore define the effect of interest as the *residual* that survives after term frequency is equalized. Alongside orig and cf, we construct a frequency-matched control (cfb) that keeps the plan-framed title but adds the criterion term to the *body*, so that cf and cfb contain the criterion term equally often and differ only in its *position* (title vs. body).

A second objection arises from the primacy bias of dense encoders [6,7]: a title is also the *earliest* text, so a title advantage might reduce to known early-position weighting. We therefore add a fourth variant (cfs) that keeps the plan title and inserts the same criterion mention as the *first line of the body*, immediately after the title. The four variants decompose the total title effect (cf−orig) into three additive components as follows: a frequency component (cfb−orig), an early-position component (cfs−cfb, the identical string moved from the end of the body to its start), and a title-framing component (cf−cfs, title versus body-start at matched frequency and near-matched position). A vocabulary-mismatch account predicts no effect beyond the frequency component; a pure primacy account predicts no effect beyond the early-position component; the framing-bias (aboutness) account predicts a residual framing effect—which is what we test in Section 5, together with a fifth control (defined in Section 4.4) that separates the following two edits that criterion-framing bundles: adding the criterion to the title and removing the plan framing. The construction also preempts the vocabulary rebuttal directly: the criterion demonstrably exists in the body of every case, so any remaining title advantage cannot be attributed to the term being merely absent elsewhere.

## 4. Materials and Methods

### 4.1. Corpus and Controlled Variables

The study uses three related indices over the Korea Expressway Corporation *Design Practice Guidelines*; Table 1 summarizes their spans, sizes, and roles. The **research corpus** (8041 chunks over 1388 unique document sections, 2005–2024) underlies the population analysis and the counterfactual probe. The **operational index** (Pinecone, 24 year-keyed namespaces) serves the Level 2 retrieval comparison of Section 5.3 (dense rankings from Pinecone; lexical rankings from a BM25 index built over the same 9434-chunk snapshot), the reframing oracle, and the prevalence study. The **retrieval-experiment index** combines all guideline chunks with a supplementary commentary collection (710 chunks), re-embedded at 1536 dimensions. Because section-level chunk identifiers recur across yearly editions, index keys are made unique per source file, preventing cross-year collisions and yielding 10,144 chunks; every gold chunk was verified against its archived source text. The rank-displacement analysis of Section 5.2 and all numbers in Section 5.7, Section 5.8, Section 5.9 and Section 5.10 are computed on this index. Ranks are comparable only within an index.

These are not three views of one collection but three collections. They differ in span, what was chunked, where the vectors were produced, and when. We therefore make no claim that rests on comparing a number from one row against a number from another, and we flag the two places where the temptation arises. The mean rank of 254.8 in Section 5.4 (operational index) and the 13.6 of Section 5.2 (retrieval-experiment index) describe the same population on different collections and are not a before/after pair; the burial rates of Section 5.5 are likewise internal to the operational index. Where the discussion draws these strands together (Section 6.1 and Section 6.3), the claims are about the *direction and mechanism* that each index independently exhibits, not about the magnitudes, which are not commensurable.

A defining property of this corpus is the rhetorical framing of section titles as follows: plan-type titles dominate (877 of 1388 sections, 63.2%), yet 86.4% of plan-titled sections contain criterion-type vocabulary in their body text (the full breakdown and token frequencies are given in Section 3.2 and Section 5.1). This high base rate identifies a large population that is structurally susceptible to a mismatch between title framing and body content, and it yields 566 sections eligible for the controlled experiment of Section 4.4.

Throughout the experiments we hold the following variables constant. Dense embeddings use OpenAI text-embedding-3-large at 1536 dimensions, indexed in Pinecone under cosine similarity. Lexical retrieval uses rank_bm25 BM25Okapi [23] with k1 = 1.5, b = 0.75, and ε = 0.25, over a Kiwi morphological tokenizer augmented with domain-specific regular expressions; the resulting vocabulary contains 60,808 types at a mean of 286.7 tokens per chunk. Chunking uses the tiktoken cl100k_base encoder. The Fixed scheme caps chunks at 1000 tokens with 150-token overlap; the Hierarchical variant segments by paragraph and table boundaries with zero overlap. For the 2024 eight-chapter subset, the Fixed scheme produces 344 chunks and Hierarchical produces 291.

### 4.2. Parsing and Ground Truth

Cell-level ground truth is extracted directly from the original HWPX XML by our HWPX parser, which recovers cell coordinates and values rather than relying on rendered output. Across the 2024 eight-chapter subset this yields 1229 tables and 12,463 cells; across the full corpus the parser recovers 26,757 tables. These totals count every extracted table, including nested ones, and every grid position. The pathway comparison of Section 5.6 scores a subset of them: non-nested tables whose cells carry text, with cell counts taken over non-empty values and nested-table placeholders excluded, which leaves 1069 tables and 8537 cells (the Level 1 preservation results reported in Section 5.6). These coordinate-anchored cell values form the basis for ground-truth answer verification.

On this foundation we construct a QA benchmark of 50 questions, each validated against the extracted cell coordinates (50/50 cell-consistent, with the gold answer-bearing chunk resolved in the corpus). Questions are semi-automatically generated from ground-truth cells and verified, spanning single-fact (22), table-extraction (17), framing-bias (9), and temporal (2) categories; further expansion and human relevance labeling are natural next steps (Section 6.6).

The same cell-coordinate ground truth supports a Level 1 *table-preservation* comparison across parsing pathways (Section 5.6). For each 2024 chapter we extract tables from the PDF rendering using a union of two engines (PyMuPDF find_tables and pdfplumber extract_tables) and from the one available HML export using a structure-aware HML parser ported from our HWPX table model (HWPML TABLE/ROW/CELL with row/column addresses). Each pathway’s recovered cell content is then scored against the HWPX ground truth as follows: we align each ground-truth table to the best-overlapping recovered table within the same document and report cell-content preservation (the share of ground-truth cell values recovered) and table-detection rate (ground-truth tables with ≥50% of cells recovered). Matching is by normalized text containment, which is deliberately lenient toward PDF (it does not penalize cell-boundary differences); a self-test scoring the ground truth against itself returns 100%, confirming the harness introduces no spurious loss. The two-engine union is likewise conservative in the direction favoring PDF; evaluating alternative extractors (e.g., Camelot, Docling), corpus-scale HML, and the HTML coordinate pathway is left to future work.

### 4.3. Pure Retrieval Harness

All evaluation runs use a *pure* retrieval harness with production-only enhancements—cross-encoder reranking, metadata filtering, and query decomposition—disabled. This isolates each retriever’s intrinsic behavior and prevents operational heuristics from confounding the comparison.

We evaluate the following three retrievers: dense (cosine over text-embedding-3-large), lexical (BM25Okapi as configured above), and a hybrid. The hybrid retriever fuses the dense and lexical rankings using weighted Reciprocal Rank Fusion (RRF) with k = 60 and a mixing weight α ∈ [0, 1] governing the relative contribution of the two ranked lists. Unless otherwise stated, hybrid results are reported at α = 0.5.

No hyperparameter in this study was fitted on the data it is evaluated on. BM25 uses the library defaults (k1 = 1.5, b = 0.75, and ε = 0.25) and RRF uses the conventional k = 60, both fixed before any evaluation run. The hybrid of Section 5.7, Section 5.8, Section 5.9 and Section 5.10 is unweighted RRF over the two channels, which is order-equivalent to α = 0.5 and is the value the production system had been deployed with; it was not selected by comparing fusion weights on the benchmark. Section 5.3 additionally reports α = 0.3 because the operational-index comparison sweeps α, and we report the full sweep rather than its maximum; the α sweep for the non-lexical set is likewise reported in full in Section 5.8 rather than at a selected operating point. This means the study has no held-out test split in the strict sense: it has fixed, pre-declared settings and full-sweep reporting, which we consider the honest description rather than a train–development–test protocol (Section 6.5).

### 4.4. Controlled Counterfactual Design

To establish a causal rather than correlational account of framing bias, we use a controlled counterfactual probe instead of observational corpus ranks. We draw *n* = 80 sections at random across all years (2005–2024) from the 566 eligible sections identified in Section 4.1.

For each section we construct six title/body variants—five primary and a sixth neutral-token control (cfn, introduced below)—over the same original body text (two of them additionally append a single frequency-matched criterion mention; Table 2).

Variant construction is fully rule-based, with no language model involved. The criterion is the first *~gijun* (“-standard”) compound that occurs in the section body but not in its title; the cf title is formed by stripping the plan-framing tokens (a fixed 17-item lexicon: 방안, 개선, 확충, …) from the original title and appending the criterion term. Note that cf therefore both adds the criterion to the title and removes the plan framing—the two edits that jointly define criterion-framing. A fifth variant cfp separates them: it keeps the plan title intact and appends the criterion term to it, so that cfp vs. cfs compares the title slot against the first body line with the plan framing retained in both, and cf vs. cfp isolates the removal of the plan framing itself (Section 5.2). Because cf deletes the plan tokens, however, it also shortens the title and raises its lexical overlap with the query, so cf−cfp still confounds plan semantics with title token composition. A sixth variant cfn separates these in turn: each plan token in the title is **replaced** by a semantically neutral noun of the same length rather than deleted, and the criterion is appended exactly as in cfp. The substitution is a fixed one-to-one dictionary declared before the run (방안 → 부문, 개선 → 영역, 검토 → 항목, 계획 → 측면, 조정 → 범위, 추진 → 부분, 제고 → 대상, and 강화 → 요소); all eight plan tokens occurring in these titles are two syllables long, all replacements are two syllables, and none of the replacements belongs to the plan or standard lexicons or occurs in any query or original title. cfn therefore holds title length, token count and criterion frequency fixed against cfp and differs from it only in whether the remaining title tokens carry plan semantics, while cf vs. cfn isolates the shortening and query-proximity that deletion introduces. Each variant is embedded as a single markdown document (## title line, blank line, body; truncated at 8000 characters). The query is formed as *topic* + *criterion*, where the topic is the stripped title, and for each variant we measure the cosine similarity cos(*query*, *variant*). The **cf vs. orig** contrast isolates the effect of moving the criterion into the title. The **cfb vs. orig** contrast measures the pure term-frequency effect. The **cfs vs. cfb** contrast moves the identical added string from the body’s end to its start and thus measures the within-body early-position (primacy) effect. The decisive **cf vs. cfs** contrast compares the title slot against the first body line at matched frequency and near-matched position, isolating the title-framing (aboutness) residual. This design preempts both standing objections at once as follows: vocabulary mismatch (the criterion vocabulary is genuinely present in the body) and reduction to known primacy bias (early position is explicitly controlled).

To confirm the finding is not an artifact of a single embedding family, we replicate the orig/cf contrast with an independent model, Cohere embed-multilingual-v3.0.

### 4.5. Metrics and Statistical Analysis

Retrieval quality is measured with Recall@10, MRR@10, and nDCG@10 over the 50-item benchmark. Two variants of mean reciprocal rank appear in this paper, and we distinguish them by name throughout. **MRR@10** truncates at rank 10: a gold chunk ranked below 10 contributes zero. It is used wherever the operational-index comparison of Section 5.3 is reported, together with the cross-encoder figures derived from it. **MRR**, without a cutoff, averages 1/rank over the full ranking, so a gold chunk at rank 40 still contributes 1/40. It is used for the non-lexical and sensor-stratum evaluations of Section 5.4, Section 5.7, Section 5.8, Section 5.9 and Section 5.10, where the low-overlap regime places many gold chunks below rank 10 and truncation would discard exactly the signal under study. Within each table all systems are scored with the same variant, so every comparison and significance test is internally consistent; the two variants are never compared across tables. For the counterfactual probe the dependent measure is the per-section change in cosine similarity between variants.

All comparisons are paired and evaluated with the Wilcoxon signed-rank test. For the three-way retriever comparison, and likewise for the six CAR method-pair comparisons of Section 5.8 (CAR vs. dense/BM25/hybrid across the full and low-overlap sets), we apply Holm correction to control the family-wise error rate; we report one-sided *p*-values throughout (reflecting the directional hypotheses; this is restated in the table captions) and add bootstrap 95% confidence intervals (2000 resamples over the QA items) so that small differences are not over-read. Effect sizes are reported as Cohen’s *d_z_* for paired designs. The statistical routines were implemented directly in NumPy and cross-validated against SciPy (scipy.stats); reported *p*-values and effect sizes match to three significant figures. Limitations of the benchmarks and designs are discussed in Section 6.5.

### 4.6. Criterial Reframing and Full-Corpus Evaluation

The framing-bias diagnosis suggests a direct, training-free remedy applied at *index time*. For a plan-framed section, we surface the criterion stated in its body in the section’s indexed header (a *criterial reframing* of the document) and then re-embed. Because the injected criterion is extracted from text the document already contains, no external information is added; the method only re-weights what is present so that dense retrieval can surface it. In the experiments reported here the injected criterion is the body’s ~기준 criterion compound, a deterministic proxy for an automatic extractor; integrating a learned extractor is left to future work.

We evaluate reframing against the full operational corpus (9434 vectors, 24 namespaces). Of the 80 counterfactual sections of Section 4.4, the 78 whose target section is present in this index snapshot form the evaluation set (the remaining two do not resolve in it). For each of the 78 framing-prone cases we measure the rank of the target document under (i) its original plan-framed indexing and (ii) its reframed indexing (the reframed header takes the full criterion framing of Section 4.4—the cf form, with the plan markers removed—precisely the edit that the fifth-variant control of Section 5.2 identifies as carrying the framing effect), holding all other documents fixed, and compare with paired Wilcoxon tests and Cohen’s *d_z_*. This *index-side* correction is complementary to the *retrieval-side* hybrid of Section 4.3 and distinct from query-side document expansion (doc2query [10], HyDE [11], Query2doc [24]), which rewrites or generates queries rather than re-framing the indexed document.

### 4.7. Reproducibility

Models are pinned as follows: dense embeddings use OpenAI text-embedding-3-large (1536 dimensions); the cross-model replication uses Cohere embed-multilingual-v3.0, and cross-encoder reranking uses Cohere rerank-multilingual-v3.0; and QA generation and the CAR criterion hypothesis use Claude Sonnet 4.6 (claude-sonnet-4-6). Software versions: Python 3.13; openai 2.33, anthropic 0.97, cohere 5.21, kiwipiepy 0.23.1 (Kiwi), rank_bm25 (BM25Okapi), PyMuPDF 1.27.2, pdfplumber 0.11.10, numpy 2.4, scipy 1.17, tiktoken 0.13, pinecone 8.1. Retrieval scoring, rank fusion, and all statistics are deterministic; the language-model steps (QA generation and the criterion and generic hypotheses) use default decoding, and their prompts are listed in the Supplementary Material (Sections S1–S3). The evaluation and analysis code, the counterfactual-probe harness, and derived artifacts sufficient to reproduce every reported statistic (per-variant cosine tables, gold-rank tables, the QA schema with cell coordinates, and the cache-only re-derivation script rank_reembed_eval.py) will be released in a public repository with a DOI. The production HWPX parser that feeds the ground-truth extraction is proprietary to the operating retrieval system and is not part of the release; its extraction behavior is specified in Section 4.2, and because the raw corpus is itself access-restricted (see the Data Availability Statement), the parser is not required to reproduce any reported statistic from the released artifacts.

## 5. Results

This section reports the framing-prone population in the corpus (Section 5.1), the controlled counterfactual probe isolating the framing-bias effect (Section 5.2), the Level 2 retrieval comparison across BM25, dense, and hybrid systems (Section 5.3), and the reframing, prevalence, parsing-fidelity, overlap-stratification, CAR, cost, and case analyses (Section 5.4, Section 5.5, Section 5.6, Section 5.7, Section 5.8, Section 5.9 and Section 5.10). Section 5.1 and Section 5.2 characterize the 8041-chunk research corpus (2005–2024; 1388 unique document sections), with the rank-displacement bridge closing Section 5.2 computed on the 10,144-chunk retrieval-experiment index; the Level 2 comparison of Section 5.3 and the reframing and prevalence studies of Section 5.4 and Section 5.5 run on the 9434-vector operational index (Section 5.3 dense rankings served by Pinecone, its lexical rankings by a BM25 index over the same snapshot), and the retrieval experiments of Section 5.7, Section 5.8, Section 5.9 and Section 5.10 on the 10,144-chunk retrieval-experiment index, all as defined in Section 4.1.

### 5.1. The Framing-Prone Population

We first characterize how Korean highway-design sections frame their titles. Classifying all 1388 sections by title vocabulary yields the following four groups: *plan*-type titles (e.g., *bangan* “plan,” *gaeseon* “improvement”) dominate at 877 sections (63.2%), *standard*-type titles (e.g., *gijun* “criterion,” *jichim* “guideline”) account for only 135 (9.7%), mixed titles for 292 (21.0%), and 84 sections (6.1%) carry neither marker. The title-token frequencies are consistent with this skew as follows: *bangan* (614), *gaeseon* (516), and *geomto* “review” (378) all outrank *gijun* (358).

**Figure 2:**
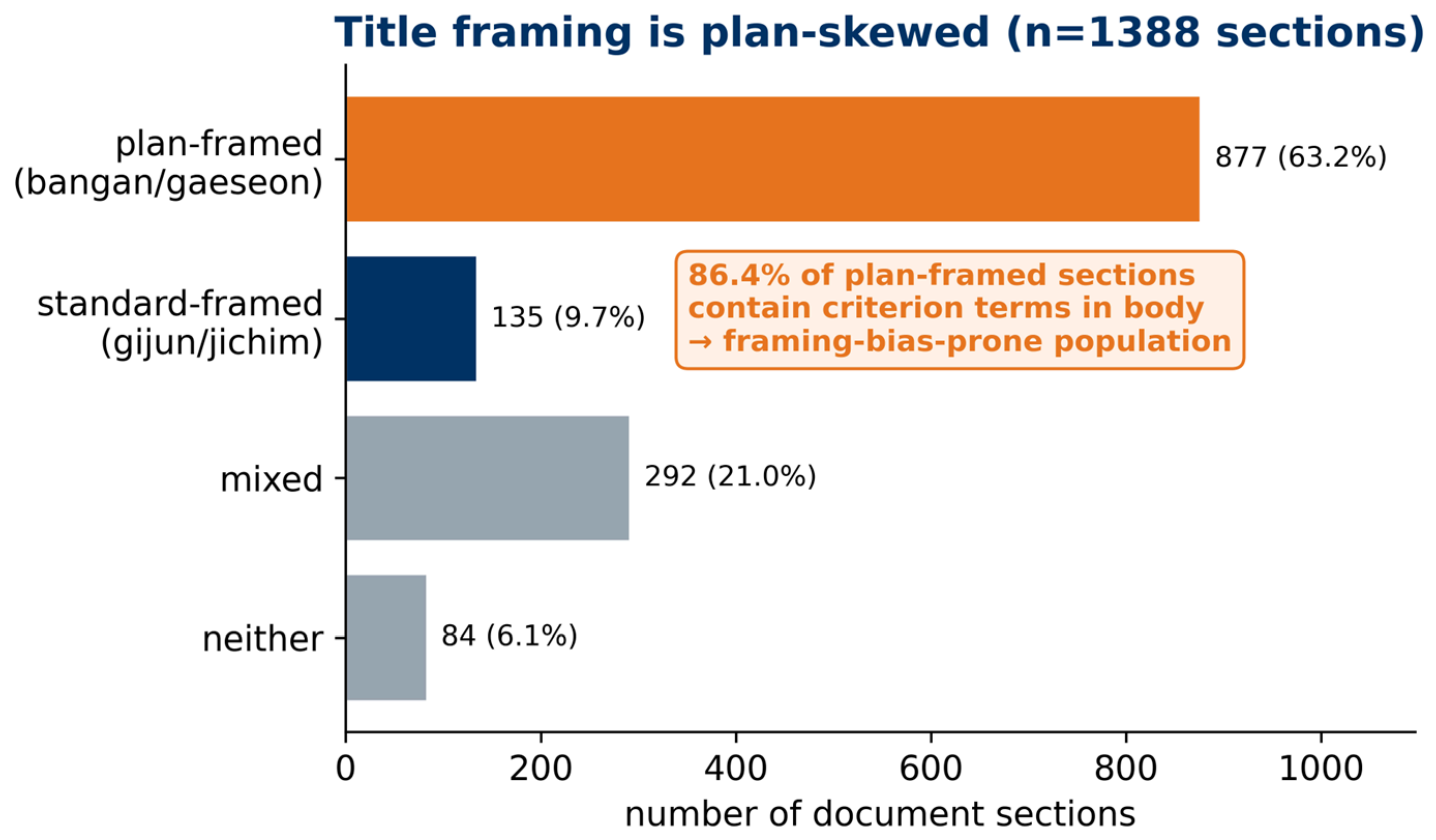
Title framing in the corpus is strongly plan-skewed (*n* = 1388 sections). Plan-framed titles (*bangan* “plan,” *gaeseon* “improvement”) dominate standard-framed titles (*gijun* “criterion/standard”); critically, 86.4% of plan-framed sections nonetheless contain criterion vocabulary in their body text, defining the framing-prone population.

The key structural fact is the mismatch between title framing and body content. Among the 877 plan-titled sections, 86.4% nonetheless contain *criterion*-type terms in their body text. In other words, the criterial content a user typically searches for is present in the body, while the title advertises a *plan*. This population is precisely the one in which an aboutness-driven retriever can be misled, and it defines the 566 sections eligible for the controlled experiment below. Figure 2 shows the plan-skewed title distribution and the criterion base rate that together define this population.

### 5.2. Controlled Counterfactual: Framing Bias

To establish causality rather than correlation, we ran a controlled counterfactual probe on *n* = 80 sections drawn at random from the 566 eligible cases across all years (2005–2024). For each section we hold the body text fixed and vary only the title or the placement of one criterion mention, in the conditions defined in Table 2—the four primary ones being *orig* (plan title, criterion in body), *cf* (criterion title), *cfb* (criterion appended at the body end, frequency-matched), and *cfs* (criterion inserted as the first body line, frequency- and position-matched); the fifth control *cfp* is analyzed at the end of this subsection. The query combines the topic with the criterion term, and we measure cosine similarity between the query and each variant.

The main contrast, *cf* vs. *orig*, shows that moving the criterion term into the title raises query–document similarity by Δ = +0.0303 in 78 of 80 cases (98%), a highly significant and large effect (Wilcoxon *p* = 1.1 × 10^−14^, Cohen’s *d_z_* = 1.63). Because *cf* changes both the frequency and the placement of the criterion term, we add the following two frequency-matched controls: *cfb* (criterion at the body end) and *cfs* (criterion as the first body line). The title wins both contrasts as follows: over *cfb* by Δ = +0.0172 (65/80, 81%; *p* = 6.7 × 10^−10^, *d_z_* = 0.90), and, in the decisive test, over the position-matched *cfs* by Δ = +0.0081 (59/80, 74%; *p* = 2.2 × 10^−5^, *d_z_* = 0.53).

The total title effect of +0.030 therefore decomposes into the following three additive, individually significant components: +0.013 term frequency (*cfb*−*orig*, *d_z_* = 1.18), +0.009 early body position (*cfs*−*cfb*, 65/80, *p* = 5.7 × 10^−9^, *d_z_* = 0.77; reproducing within this corpus the primacy bias reported for dense encoders [6,7]), and +0.008 for the title framing itself (*cf*−*cfs*), the aboutness residual that neither frequency nor position explains. To rule out an OpenAI-specific artifact, we replicated the main contrast with an independent embedding family, Cohere embed-multilingual-v3.0, which reproduced the effect at Δ = +0.0206 in 98% of cases (*p* = 9.3 × 10^−15^, *d_z_* = 1.64).

**Title slot or framing content?** Because cf bundles two edits—adding the criterion to the title and removing the plan framing—we ran the fifth-variant control (cfp: plan title retained, criterion appended to it; Table 2; Supplementary Table S6) in a single six-text embedding batch (the query plus the five primary variants) that reproduces the corresponding Table 3 Δs within 0.001. The result is unambiguous. With the plan framing retained, the title slot confers no advantage over the first body line (cfp vs. cfs: Δ = −0.0004, 40/80, n.s.), while removing the plan framing carries the entire residual (cf vs. cfp: Δ = +0.0087, 64/80, *p* = 1.1 × 10^−9^, *d_z_* = 0.83). The residual is therefore not positional: a criterion token appended to a still-plan-framed title gains nothing over the same mention at the body’s start.

**Plan semantics or title token composition?** The cf−cfp contrast still bundles two things, because deleting the plan tokens both removes plan semantics and shortens the title toward the query. The sixth variant cfn (Section 4.4) separates them by substituting neutral tokens of equal length instead of deleting. In a single six-text batch that reproduces every Table 3 contrast among its embedded variants within 0.001 (the cfb rows belong to the primary five-variant batch; Section S7), the residual divides almost evenly and both halves are significant on OpenAI: removing plan semantics at fixed length and composition contributes Δ = +0.0048 (cfn vs. cfp, 53/80, *p* = 1.3 × 10^−4^, and *d_z_* = 0.46), and the shortening that deletion introduces contributes a further Δ = +0.0040 (cf vs. cfn, 54/80, *p* = 8.8 × 10^−5^, and *d_z_* = 0.46). The two sum to this batch’s +0.0088 for cf−cfp.

The cross-model picture is mixed, and we report it as such. On Cohere the token-composition component replicates and is in fact larger (cf−cfn Δ = +0.0045, 66/80, *p* = 1.1 × 10^−8^, and *d_z_* = 0.76), but the plan-semantics component does not (cfn−cfp Δ = −0.0005, 38/80, and *p* = 0.50, n.s.). We also correct a claim made in an earlier version of this analysis as follows: on Cohere the cfp and cfs variants receive *identical* embeddings for all 80 sections, because they differ only in whether a space or a newline separates the appended criterion from the title, and that model’s tokenizer normalizes the distinction away. The Cohere cfp ≈ cfs result therefore carries no information and is not evidence for the framing account; for the same reason Cohere’s cf−cfp and cf−cfs are the same contrast, not two.

What survives, then, is narrower than a single-cause attribution. The total title effect is large and cross-model (Table 3), and its decomposition into frequency, early position and a title component is unchanged. Within the title component, the part attributable to title token composition replicates on both embedding families; the part attributable to plan semantics is significant on text-embedding-3-large and absent on embed-multilingual-v3.0. Whether that difference reflects genuine model-specific sensitivity to administrative framing or the shorter effective context of the Cohere encoder is not settled by these data. Full numbers for both models are given in the Supplementary Material (Table S7).

**Figure 3:**
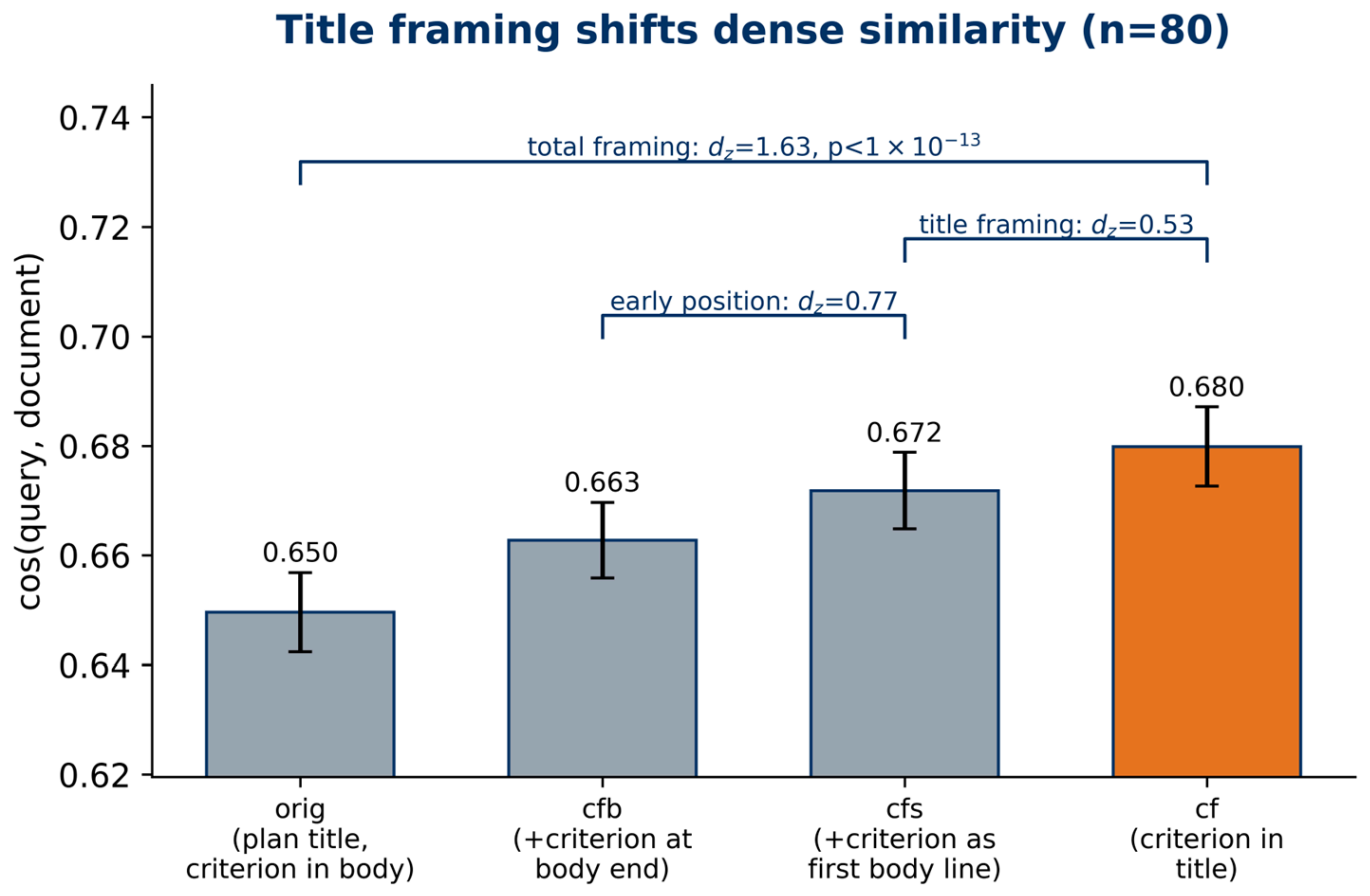
Title framing shifts dense query–document similarity (*n* = 80). Bars show mean cosine similarity (±SEM) between the criterion query and the four variants of the *same* body text (Table 2): orig (plan-framed title, criterion in body only), cfb (one criterion mention added at the body end, frequency-matched, plan title retained), cfs (the same mention moved to the first body line), and cf (criterion moved into the title). Brackets mark three of the contrasts in Table 3 as follows: the total framing effect cf−orig (*d_z_* = 1.63, *p* = 1.1 × 10^−14^), the early-position (primacy) component cfs−cfb (*d_z_* = 0.77, *p* = 5.7 × 10^−9^), and the title-framing (aboutness) residual cf−cfs (*d_z_* = 0.53, *p* = 2.2 × 10^−5^) that persists at matched frequency and near-matched position.

These results show that the criterion term contributes more to dense similarity when it frames the title than when it sits anywhere in the body at equal frequency—and that the advantage is specifically one of framing content, not of the title position as such. The effect is directionally consistent across nearly all sections, large in total magnitude, and reproducible across two independent embedding families, supporting a causal interpretation of title aboutness being over-weighted relative to body containment, over and above the known primacy weighting of early text. Figure 3 visualizes the conditions and the decomposition of the total title effect into frequency, early-position, and title-framing components. Section 5.10 makes the same effect concrete on four individual sections, with the target’s full-corpus rank before and after the criterion is surfaced.

**Figure 4:**
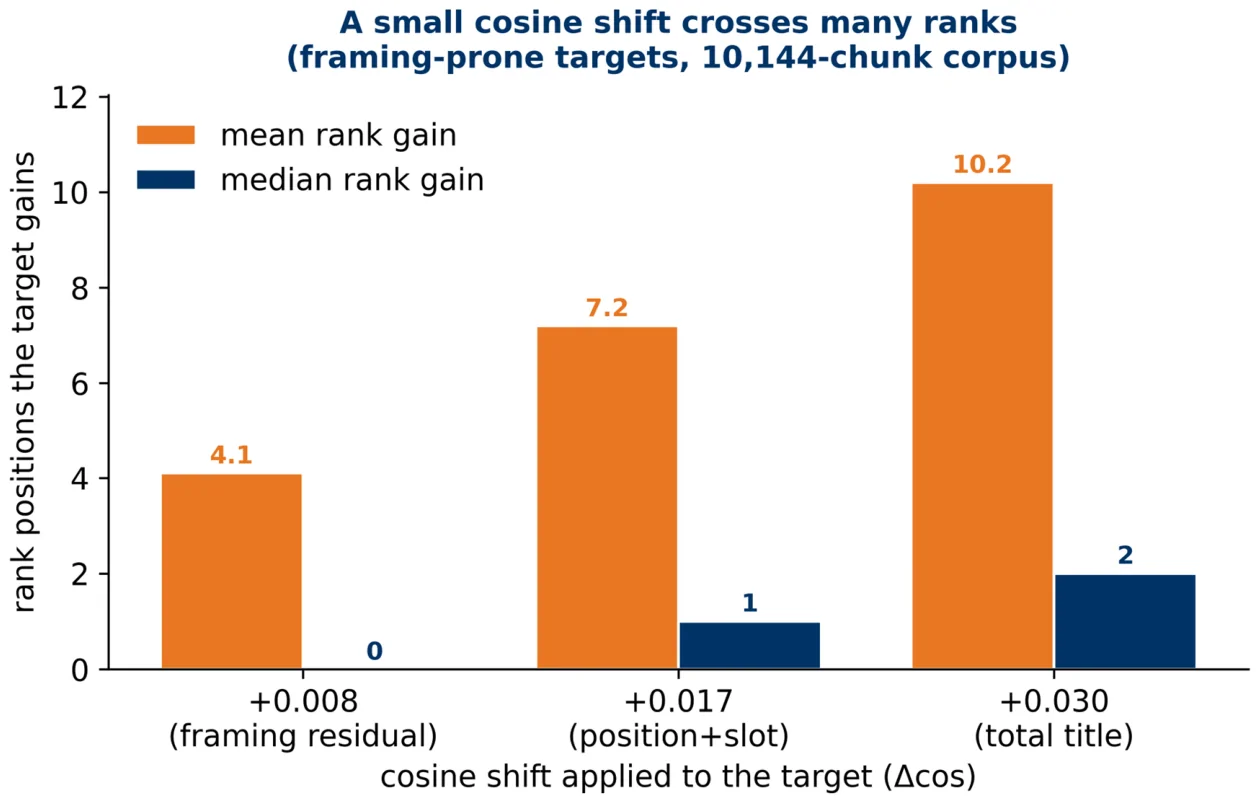
Why a small cosine shift matters (*n* = 80 framing-prone targets, 10,144-chunk retrieval-experiment index). Adding the measured title-framing cosine shifts to a target’s similarity score (an additive-constant perturbation, not a re-embedding) raises its rank by a mean of 4.1/7.2/10.2 positions for Δcos = 0.008 (framing residual)/0.017 (position + slot)/0.030 (total title effect); the small medians (0/1/2) against larger means reveal a heavy tail of buried targets that jump dozens of ranks. Because the median target sits only 0.023 cosine below rank 1, the total title effect spans the entire gap to the top.

**From cosine shift to rank displacement.** The counterfactual effect, though highly significant, is small in absolute cosine (Δ ≈ 0.01 to 0.03). It matters because corpus similarity scores are densely packed near each target as follows: on the retrieval-experiment index, the median cosine gap between a target and the top-ranked document is only 0.0231, so the total title effect spans the entire distance from the median target to rank 1. Adding the measured shifts to a target’s score raises its rank by a mean of 4.1/7.2/10.2 positions for the framing-residual, position-plus-slot, and total effects (medians 0/1/2; Figure 4), and actually re-embedding the criterion-framed variant lifts the target from a mean rank of 13.6 to 4.5 (median 3.5 to 1), improving 51 of 80 cases and worsening 1. The small medians against larger means are a heavy-tail signature: most targets already sit near the top, but the buried minority (14 of 80 at rank ≥15, down to rank 200) jumps dozens of ranks when the criterion reaches the title (200 → 56, 41 → 2). Targets of the same population sit far deeper on the operational index (mean rank 254.8; Section 5.4); that baseline is not comparable to this one, since the operational snapshot differs in chunk composition and embedding provenance (production-time vectors rather than this section’s controlled re-embedding), which is why every analysis reports ranks within a single index only (Section 4.1). For the framing-prone minority that motivates this paper, a 0.02–0.03 cosine penalty is the difference between the first page of results and the tail.

### 5.3. Level 2 Retrieval Comparison

We next evaluate retrieval quality on the 50-item QA benchmark (cell-coordinate ground truth) using a pure retrieval harness with all operational reranking and boosting disabled (Section 4.3), on the operational-index snapshot of Section 4.1 (dense rankings served by Pinecone; BM25 built over the same snapshot). Table 4 reports Recall@10, MRR@10, and nDCG@10 for BM25 (rank_bm25 BM25Okapi over a Kiwi morpheme tokenizer), dense retrieval (text-embedding-3-large, Pinecone cosine), and weighted-RRF hybrids at two fusion weights (α = 0.3 and 0.5).

Dense retrieval is significantly weaker than BM25 across all three metrics as follows: MRR@10 drops by Δ = −0.322 (Holm-corrected *p* = 1 × 10^−4^, *d_z_* = −0.75), nDCG@10 by Δ = −0.301 (Holm *p* < 10^−4^, *d_z_* = −0.85), and Recall@10 by Δ = −0.205 (Holm *p* = 0.002, *d_z_* = −0.55). The hybrid recovers a significant part of this gap (best at α = 0.3, where the lexical channel carries more weight), improving over dense on nDCG@10 by +0.150 (Holm *p* = 0.002, *d_z_* = 0.57), MRR@10 by +0.109 (Holm *p* = 0.029), and Recall@10 by +0.166 (Holm *p* = 0.006). The hybrid does *not*, however, beat BM25 here: because the QA answers are cell values that match lexically, the task favors exact-term retrieval, and BM25 remains the strongest single system on this benchmark.

A natural objection is that a cross-encoder reranker, standard practice for repairing dense retrieval, would close the gap. We tested this directly, reranking dense’s top 50 candidates with Cohere rerank-multilingual-v3.0. The reranker substantially recovers *ranking quality* as follows: MRR@10 rises from 0.417 to 0.748 (matching BM25’s 0.740) and nDCG@10 from 0.476 to 0.762. However, Recall@10 rises only to 0.892, still short of BM25’s 0.962, and this residual is the crux. A reranker can only reorder the candidate pool; the criterion documents that framing bias buries *beyond* the pool (the tail quantified in Section 5.5) are never seen by the reranker and so cannot be recovered. Reranking therefore mitigates the symptom (poor ordering of retrieved candidates) but not the cause (criterion documents excluded from retrieval); the index-side correction of Section 5.4 addresses the cause directly by surfacing the buried document into the candidate set. Section 5.8 tests this asymmetry rather than leaving it as an inference: on the non-lexical set the reranker again recovers ranking while its recall stays pinned to that of its input channel.

**Figure 5:**
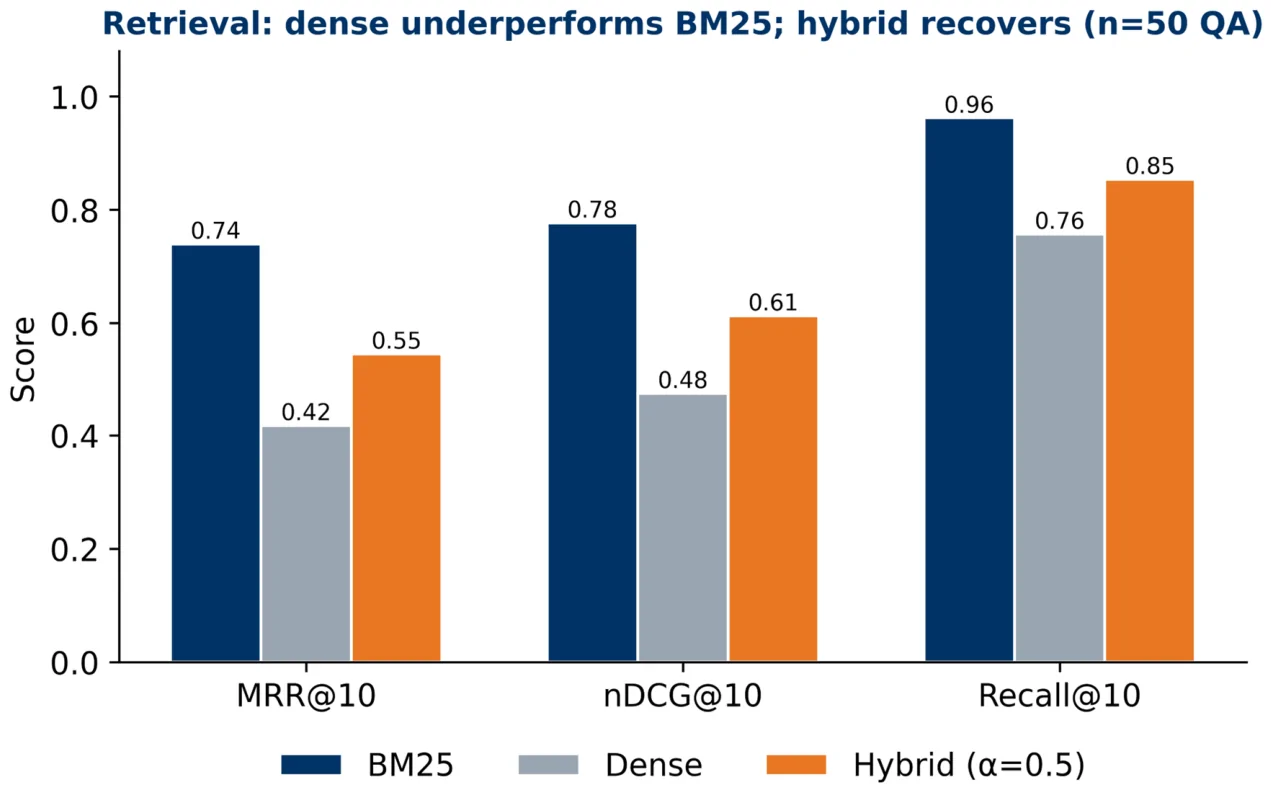
Level 2 retrieval on the QA benchmark (*n* = 50). Dense retrieval (text-embedding-3-large) underperforms BM25 on the rank-sensitive MRR@10 and nDCG@10; the weighted-RRF hybrid (α = 0.5) partially recovers over dense. BM25 remains the strongest single system here because the QA answers are lexically matched cell values: a caveat discussed in Section 6.3.

On the framing-bias QA subset (*n* = 9; exploratory, reported descriptively only), dense is again the weakest system (MRR@10 0.559) and BM25 the strongest (0.856), with the hybrid in between (MRR@10 0.667 at α = 0.3). The ordering is directionally consistent with dense being the most hurt by title framing, but with nine items the subset is underpowered for a formal test; a larger, human-validated framing-bias QA set is needed before drawing inferential conclusions from it (Section 6.5). Figure 5 summarizes the three systems across the rank-sensitive metrics.

### 5.4. Criterial Reframing: Oracle Ceiling and the Limits of Index-Time Correction

**Figure 6:**
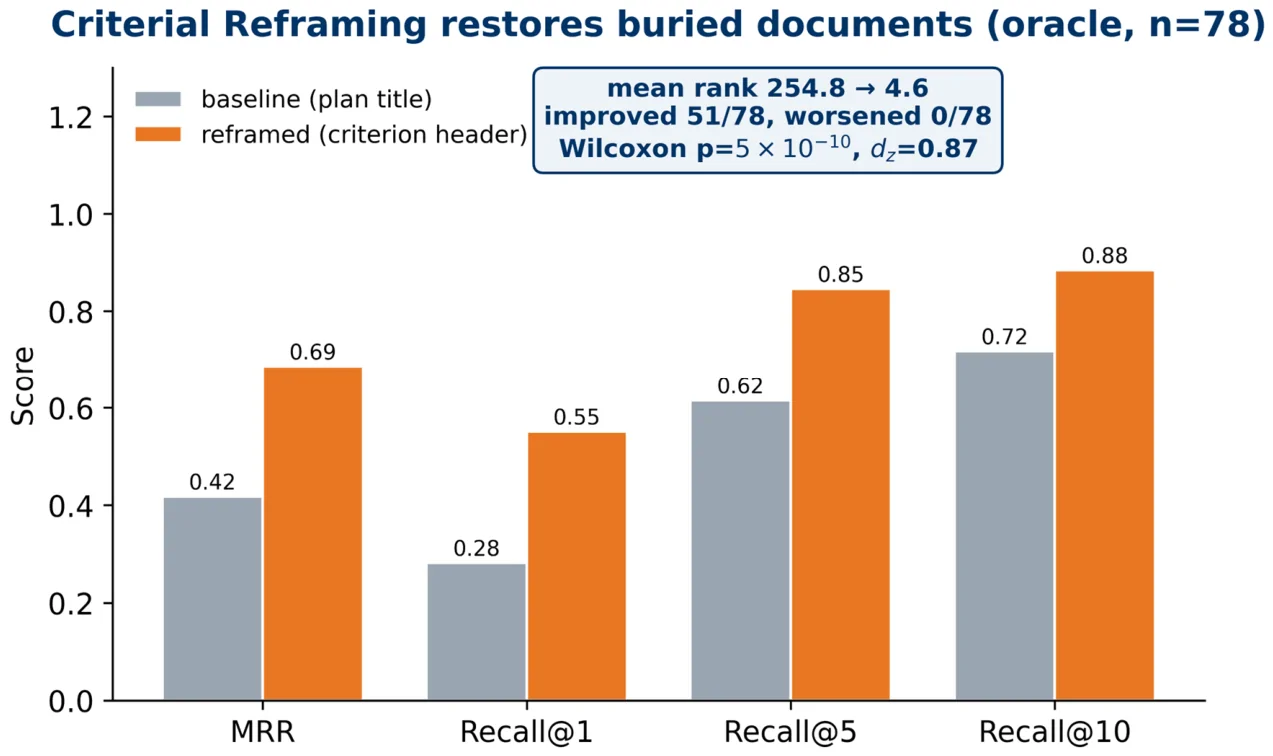
Index-time Criterial Reframing on full-corpus retrieval (*n* = 78), **oracle condition** (the queried criterion is injected). Reframing moves the target from a mean rank of 254.8 to 4.6 (improving 51/78, harming none), establishing the mechanism’s achievable ceiling. A deployable variant with query-independent extraction does not transfer to end-to-end QA (Section 5.4); this figure bounds what reframing could achieve, not what naive automation delivers.

The framing-bias diagnosis points to a direct remedy at index time (Section 4.6). Evaluated against the full 9434-vector corpus over the 78 framing-prone cases, criterial reframing is large in effect and uniformly beneficial (Table 5, Figure 6). It cuts the target document’s mean rank from 254.8 to 4.6 and its median rank from 4 to 1; MRR rises from 0.418 to 0.686 and Recall@10 from 71.8% to 88.5%. The correction improved retrieval in 51 of 78 cases and worsened it in none (Wilcoxon *p* = 5.2 × 10^−10^, Cohen’s *d_z_* = 0.87).

The baseline mean rank of ~255 is itself a finding: in realistic full-corpus retrieval, the criterion-bearing targets of this framing-prone population sit deep in the ranking—an observation consistent with, though not by itself attributable to, the causal effect isolated in Section 5.2 (and with the motivating case of Section 1, where the target sat at rank 78 of ~1500). Under this oracle condition, reframing essentially undoes the displacement and harms no case, but, as we show next, this reflects the mechanism’s *ceiling* rather than a deployable guarantee.

**Oracle ceiling vs. deployable extraction.** The evaluation above injects the criterion that the query itself targets, an *oracle* condition that establishes the mechanism’s *ceiling* (what reframing achieves when the right criterion is surfaced), not a turnkey method. We therefore tested a deployable variant on the QA benchmark: a query-independent extractor (the first body criterion compound) reframes each gold section by appending the extracted criterion to its existing header, and we re-score against the corpus using the *independent* QA questions rather than criterion-derived queries. The extractor covers 66% of gold sections (others state no extractable criterion compound), and on these reframing does *not* transfer: MRR moves 0.440 → 0.375 (6/33 improved, 12/33 worsened; Wilcoxon *p* = 0.093, n.s.). We further tested richer query-independent extractors (injecting *all* distinct body criteria, or only the *most frequent*) and none transferred either (MRR 0.383–0.389, all n.s.). The limitation is therefore *structural*, not a tuning artifact: an index-time reframer cannot anticipate which of a section’s several criteria a given query will seek, so it injects as much noise as signal. This is an informative negative result. It relocates the correction to the *query-aware* side of the pipeline, exactly where lexical retrieval operates, matching the body’s criterion term to the query directly. The oracle ceiling marks what is achievable *with* query knowledge; the dependable remedy on this corpus is to keep a lexical (BM25) channel in the loop, which is precisely why BM25 (and the hybrid) outperform dense here. Index-time correction of framing bias is thus fundamentally limited.

**Index-side baselines: is the title itself the problem?** The oracle above rewrites the title’s *content*; a simpler family of remedies manipulates its *presence* in the index: retrieving on the body alone (title removed), embedding title and body separately and mixing the two similarities with a weight *w*, fusing the title-only and body-only rankings late (reciprocal-rank fusion), and max-pooling the two similarities per chunk. We implemented these four remedies plus a whole-chunk control—five configurations in all—on the same 10,144-chunk index, with the title/body split fixed by rule before the run (the leading contiguous heading lines of a chunk are its title, the remainder its body; all 10,144 chunks resolve under the rule), and evaluated them on the combined 131-query benchmark (the 50 lexical and 81 non-lexical items) stratified by overlap; the whole-chunk control reproduces the archived dense channel exactly on Recall@10 (0.687) and to 0.002 on MRR (0.341 vs. 0.339), validating the harness. The full grid is Supplementary Table S10. No configuration matches the whole-chunk control overall (the best alternative, max-pooling, posts MRR 0.301 against 0.341 and Recall@10 0.550 against 0.687), and on the low-overlap stratum—where the diagnosed bias concentrates and where CAR claims its advantage—every alternative is worse than leaving the chunk intact (best 0.183 against 0.229). The field-weighted arm was swept over the full grid *w* ∈ [0, 1] with no operating point matching the control overall, and removing the title outright costs 0.098 MRR and 0.183 Recall@10. Three of the tabulated configurations edge the control on the mid stratum by ≤0.016, and max-pooling also edges it on the lexical stratum (0.436 against 0.424; Table S10); none of these affects the overall or low-overlap ordering, and we attach no claim to the differences. The reading is the one the diagnosis predicts: the title is not noise to be deleted but signal that is mis-weighted in a particular regime, so index-wide deletion or re-weighting trades the low-overlap failure for a larger loss everywhere else, and the correction has to be query-aware. A second evaluation surface (the 80 framing-prone targets) is reported in Table S10 under a construct caveat as follows: its queries are derived from section titles (Section 4.4), so title-heavy configurations dominate it by construction and that surface cannot rank retrieval quality.

### 5.5. Observational Prevalence: Does the Bias Surface in Practice?

The counterfactual (Section 5.2) establishes a causal title-framing effect under controlled rewriting; we next ask how often it surfaces in *unedited* retrieval. We compare two groups of real sections that each state a criterion in their body, plan-framed (*n* = 78) versus standard-framed (*n* = 88), issuing a criterion query for each and recording the rank of its source document in full-corpus dense retrieval (relevance operationalized as retrieval of the source document; human relevance labels remain future work).

The effect manifests as a *tail risk* rather than a uniform shift (Table 6). Median ranks are similar (plan 4, standard 3) and the full-distribution difference is only marginal (Mann–Whitney *p* = 0.058). However, the *burial rate* (how often the criterion-bearing document falls outside the usable top-*k*) is significantly higher for plan-framed sections: 27% vs. 12% beyond rank 10 (Fisher’s exact *p* = 0.015, OR = 2.6) and 12% vs. 3% beyond rank 50 (*p* = 0.042, OR = 3.7); the two cutoffs correspond to natural deployment depths, the first page of results and a typical rerank-pool boundary. Across the three correlated tests reported here (the full-distribution Mann–Whitney and the two burial-rate comparisons), Holm correction leaves only the beyond-rank-10 difference significant (*p*_Holm = 0.045); the beyond-rank-50 difference (*p*_Holm = 0.084) and the distributional test (*p* = 0.058) do not survive, so we read the deep-tail (>50) result as exploratory and consistent with the marginal omnibus. Framing bias thus does not degrade the typical query; it creates a heavy tail of badly buried criterion documents, the very cases in which an engineer or digital-twin module silently misses the governing standard. This observational prevalence complements the controlled causal estimate of Section 5.2, with the caveat that the two groups differ in topic as well as framing, a confound the counterfactual avoids by construction. To check that the source-document relevance proxy is not itself broken, an independent LLM relevance audit judged, for every one of the 170 query–document pairs, whether the source document actually states the queried criterion (the 170 pairs are the full pre-exclusion pool of 80 plan-framed and 90 standard-framed queries; the rank comparison above retains the 166 whose source document is retrieved within the top 1000): it was judged relevant in 100% of plan-framed and 93% of standard-framed pairs (the few standard-framed exceptions are incidental criterion-term matches), supporting the positive side of the operationalization. This audit does not replace human judgment; a two-annotator human-labeled subset with inter-annotator agreement (weighted κ) is in preparation and is the proper validation (Section 6.6).

### 5.6. Format Fidelity: Table Preservation Across Parsing Pathways (Level 1)

The bias results above concern *indexing and retrieval*; this subsection establishes the *ingestion* layer beneath them (how much tabular content each parsing pathway preserves in the first place), which both justifies the HWPX-derived ground truth and quantifies what format choice costs. We score the recovered cell content of each pathway against the HWPX cell-coordinate ground truth on the 2024 corpus (Section 4.2). As a harness check, scoring the ground truth against itself returns 100.0% (Table 7, HWPX row), confirming that the metric attributes loss to the pathway, not to the scorer.

**Figure 7:**
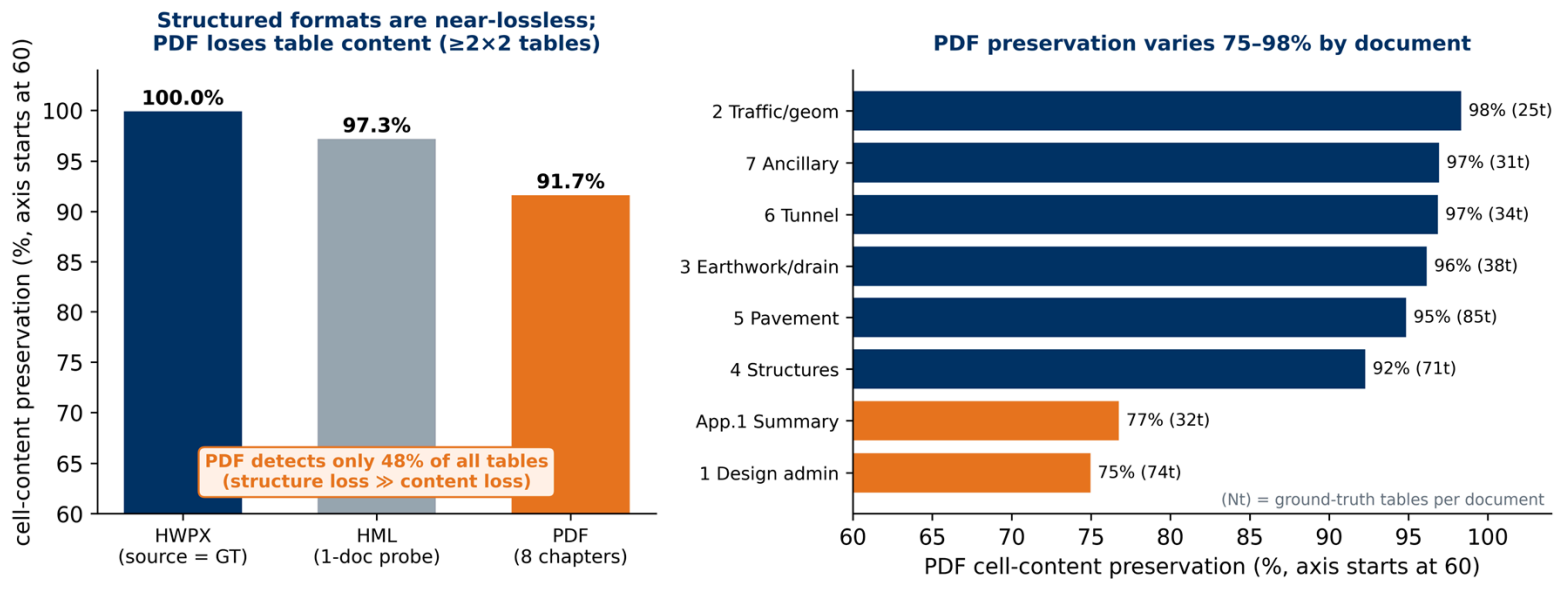
Level 1 table preservation against HWPX ground truth (2024 corpus). (**left**) cell-content preservation of ≥2 × 2 tables: structured formats (HWPX, HML) are near-lossless, while PDF both loses content and, across all tables, detects under half of them as tables. (**right**) PDF cell preservation per document, varying from 75% to 98%; the layout-heavy administration and summary documents (orange) are the weakest, not the structures chapter. Axes start at 60% to resolve the top range; “(Nt)” marks each document’s ground-truth table count. HML reflects the one document available for export; corpus-scale HML and the HTML pathway require Hangul re-export.

The structured formats are near-lossless. HWPX, being the source from which ground truth is derived, defines the 100% reference. The HML export (a single available document; chapter 1; 74 tables of size ≥2 × 2) preserves 97.3% of ≥2 × 2 cell content with full table detection, confirming that a faithful structured export loses almost nothing. PDF is markedly different. Across all eight chapters it recovers 91.7% of ≥2 × 2 cell *content*, but this figure understates the loss in two ways visible in Table 7 and Figure 7. First, *detection* collapses on small tables as follows: across all 1069 ground-truth tables PDF detects only 47.6% with ≥50% of their cells recovered, against ~100% for the structured formats. PDF’s failure is less about garbling cell text than about not recovering a table as a table at all. Second, preservation is *document-dependent*, ranging from 75.0% (design administration) and 76.8% (summary appendix) to 98.4% (traffic and geometry); the worst documents are not, as a prior tool-specific estimate had suggested, the structures chapter (here 92.3%), but the layout-heavy administrative and summary chapters. The content figure is robust to matching strictness (91.4% when single-character cells are excluded, 90.4% when cells shorter than three characters are excluded) and bimodal at the table level: of 390 ≥ 2 × 2 tables, 247 are recovered essentially perfectly (≥99% of cells) while 18 are lost entirely (0% recovered).

Two caveats bound this Level 1 result. The HML arm is a single-document proof of concept, because only one HML export currently exists; the consistent near-lossless reading is expected for a structured export but is not yet a corpus-scale claim. Also, our table alignment is one-to-one within a document, so a ground-truth table that a PDF engine splits across pages or merges with a neighbor is scored as partial loss rather than re-stitched. The takeaway is nonetheless clear and converges with the retrieval story: the format in which a criterion table is ingested measurably governs whether its cells survive to be indexed at all, and PDF, despite being the most common exchange format, is—as measured with this one extractor pair (PyMuPDF + pdfplumber; alternatives such as Camelot or Docling are unmeasured)—the pathway that most often drops them, especially for the small, layout-embedded tables in which engineering criteria frequently sit. As in Section 1, this is a claim about one pathway and one extractor pair, not a ranking of four formats.

### 5.7. When Does Dense Retrieval Help? A Lexical-Overlap Stratification

The Level 2 comparison (Section 5.3) found dense retrieval underperforming BM25, but its QA answers are cell values, an intrinsically lexical target. One might therefore suspect that dense’s deficit is a benchmark artifact. We test this directly by constructing a *non-lexical* QA set and stratifying by query–document lexical overlap.

We generated paraphrased and inferential questions whose wording deliberately avoids the source passage’s distinctive terms (e.g., asking about “the joint that expands and contracts with temperature” rather than naming the *expansion joint*), each with an answer grounded in a single source chunk. Every candidate was adversarially validated against its source passage by a separate LLM review pass for answer-groundedness and fairness; **81 of 124 candidates** survived (the rest reused source terms, were ambiguous, or attached a rationale absent from the source). The retained items were then screened by a deterministic grounding check (verbatim quote and answer-value matching against the gold chunk), and every machine-flagged item was human-reviewed by the domain-expert author (Supplementary Material S5): two items (2.5%) carried genuinely ungrounded premises and were corrected to the source’s wording before final scoring, while the remainder proved grounded once their evidence excerpts were extended verbatim from the source chunk. On the full 10,144-chunk index (Section 4.1) we then measured the rank of the gold chunk under BM25 (Kiwi tokenizer) and dense retrieval (text-embedding-3-large), binning questions by their measured lexical overlap with the source. The bin boundaries (0.35, 0.50) were fixed at benchmark construction, before any retrieval comparison; they yield near-equal strata and are held constant across all experiments.

**Figure 8:**
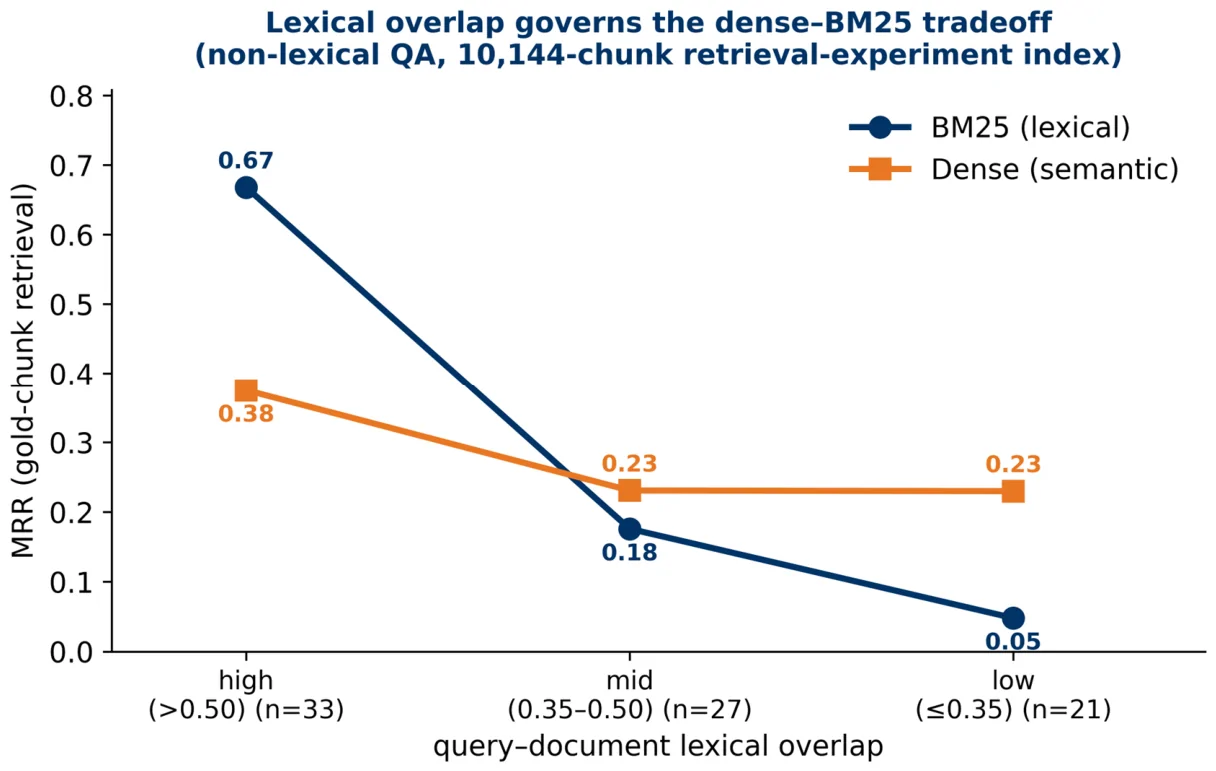
Lexical overlap governs the dense–BM25 tradeoff on non-lexical QA (*n* = 81; 10,144-chunk index). As query–document lexical overlap decreases, BM25 (lexical) collapses from MRR 0.67 to 0.05 while dense (semantic) degrades far more gently (0.38 → 0.23); dense overtakes BM25 once overlap falls below ≈0.5. Neither channel is strong at low overlap, which is the empirical case for a hybrid that retains both.

The result is a clean monotonic crossover (Table 8, Figure 8). At high overlap (>0.50) BM25 dominates (MRR 0.668 vs. 0.375); as overlap falls, BM25 collapses: 0.176 at mid overlap and then 0.048 at low overlap (≤0.35), where the gold chunk sits at a median rank of 166, while dense degrades far more gently (0.375 → 0.231 → 0.230). The crossover is reached already in the mid band (0.35–0.50), where dense edges ahead (0.231 vs. 0.176), and at ≤0.35 dense leads decisively (MRR 0.230 vs. 0.048; Recall@10 0.476 vs. 0.143). Two conclusions follow. First, dense’s Section 5.3 deficit is *not* a fixed benchmark artifact but an overlap-dependent effect: which channel wins is governed by how lexically a query restates its answer. Second (and decisive for our prescription), *neither channel is robust across the spectrum*: BM25 fails when wording diverges, and dense, while the channel that retains signal there, is itself modest (Recall@10 0.476). This is the empirical case for keeping both channels in a hybrid as a necessity rather than a tie-breaker, because the criterion-seeking queries most exposed to framing bias (Section 5.2) are exactly the low-overlap queries where a lexical channel alone fails. To state the probe’s scope, the QA items are LLM-authored, LLM-adversarially validated, deterministically grounding-checked, and author-reviewed on every flagged item (full-set human verification remains future work; Section 6.6), and the test uses a local 1536-dimension index rather than the operational store, so it is a controlled comparison of the two channels, not a re-estimate of Section 5.3’s absolute values.

### 5.8. Criterion-Aware Retrieval: A Working Query-Aware Remedy

Section 5.4 showed that index-time reframing fails because it is query-blind. **Criterion-Aware Retrieval (CAR)** closes that gap at *query* time. Given a query, a language model generates a short *criterion hypothesis*, rewriting the (often paraphrased) query into the formal criterion vocabulary a standard would use (“the joint that expands and contracts with temperature” → “expansion joint; waterproofing installation criterion”), without seeing the answer. CAR then fuses, by weighted RRF (k = 60), (i) a dense ranking of the query concatenated with its criterion hypothesis and (ii) a BM25 ranking of the criterion-expanded query. Unlike generic query expansion (doc2query [10], HyDE [11]), the hypothesis is targeted at the criterion the diagnosed bias buries; unlike index-time reframing, it is conditioned on the actual query.

**Figure 9:**
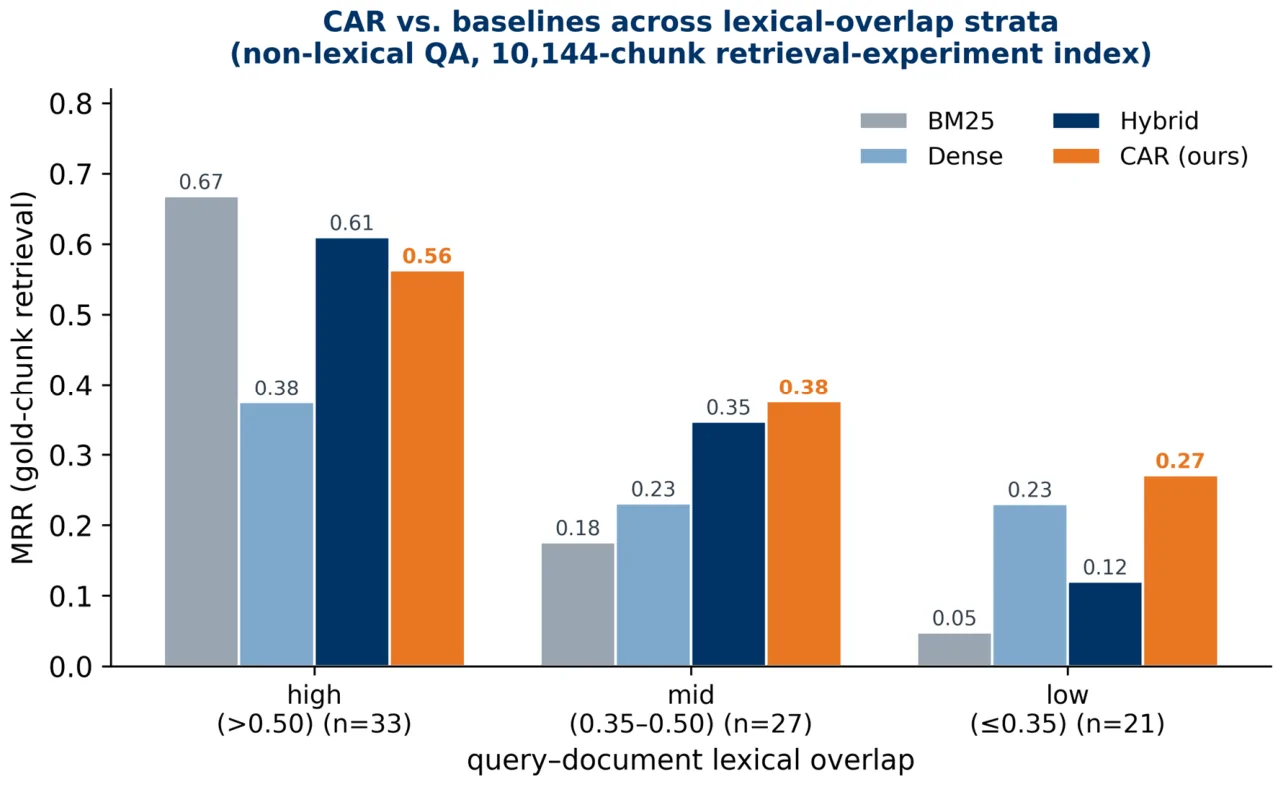
CAR (orange) versus baselines across overlap bins (*n* = 81; 10,144-chunk index). BM25 leads at high overlap; CAR leads at mid overlap and, at the low overlap where the framing bias concentrates, lifts MRR from the hybrid’s 0.120 to 0.271, a gain that survives Holm correction (*p* = 0.035), as does its low-overlap gain over BM25 (*p* = 0.005). Aggregate margins over BM25 and the hybrid do not survive correction (Section 5.8): CAR is a targeted remedy for the bias-critical regime, not a universal winner.

On the expanded non-lexical set (*n* = 81; 124 candidates adversarially validated, 81 kept), evaluated on the 10,144-chunk index, CAR shows a sharp and theoretically coherent profile (Table 9, Figure 9). *In the low-overlap regime where the diagnosed bias concentrates (≤0.35, n = 21), CAR beats both the lexical channel and the hybrid after Holm correction across the six method-pair tests*: over BM25 by +0.223 (MRR 0.271 vs. 0.048, Holm *p* = 0.005) and over the weighted-RRF hybrid by +0.151 (0.271 vs. 0.120, Holm *p* = 0.035; improving 14 cases and worsening 4). Over the full non-lexical set, CAR significantly exceeds dense retrieval (+0.136, Holm *p* = 0.001), while its aggregate margins over BM25 (+0.082, Holm *p* = 0.062) and the hybrid (+0.030, Holm *p* = 0.24) do not survive correction; the bootstrap 95% CIs overlap (CAR 0.425 [0.344, 0.507] vs. hybrid 0.395 [0.313, 0.476]).

**A stronger baseline: cross-encoder reranking.** Those contrasts, however, omit the standard repair that Section 5.3 showed to be the most effective one on the lexical benchmark. We therefore ran it here as well, applying rerank-multilingual-v3.0 to the top 50 candidates of the dense and of the hybrid channel (Table 9). On ranking quality the reranker is the better method, and by a clear margin: at low overlap Dense+rerank reaches MRR 0.376 against CAR’s 0.271, and over the non-lexical set 0.480 against 0.425. It achieves this without a language-model call and without CAR’s ~3.7 s of added latency. Any reading in which CAR is the best available remedy for low-overlap criterion queries is therefore not supported, and we do not make it.

What the reranker does not do is change which documents are available to be ranked. Because it reorders a fixed candidate pool, its Recall@50 is exactly that of the channel feeding it—0.714 at low overlap, identical to dense alone—whereas CAR reaches 0.857 there and 0.938 over the non-lexical set. This is the same asymmetry Section 5.3 reported on the lexical benchmark (ranking recovered, recall not), now reproduced on the non-lexical set and in the regime the diagnosis singles out. The two methods act on different failure modes as follows: reranking corrects the *order* of what was retrieved, while CAR widens *what is retrieved at all*, which is the failure the framing bias produces. On the low-overlap stratum the recall difference amounts to three of twenty-one questions, so we report it as a consistent direction rather than a tested effect.

The practical consequence is a revision of our earlier framing. CAR is not a replacement for reranking and does not dominate it; the two are complementary, and a deployment that cares about not missing the governing clause would widen the pool first and reorder it second. We return to this in Section 6.3.

**Figure 10:**
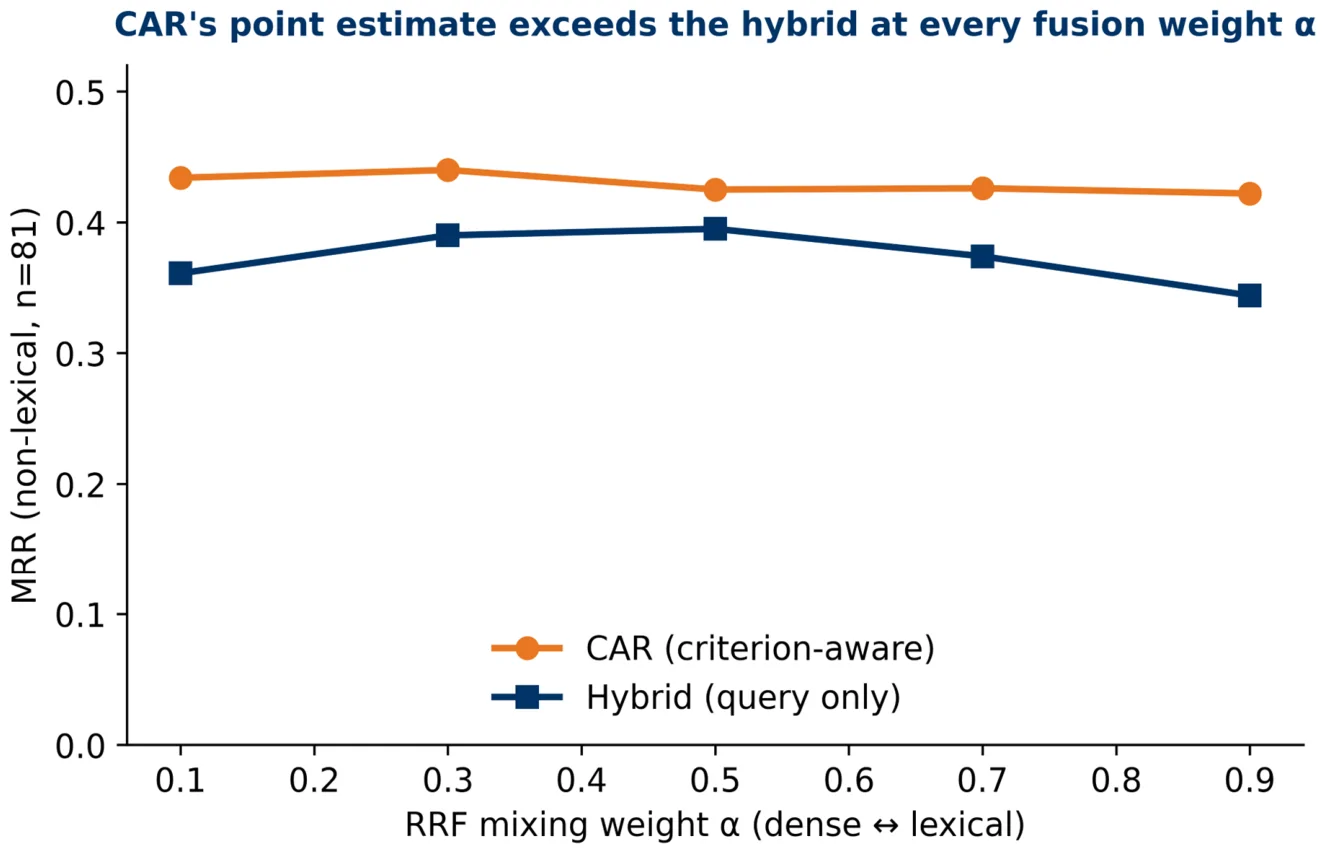
Robustness of CAR to the RRF weight α, where α weights the dense channel and 1−α the lexical channel (non-lexical, *n* = 81; 10,144-chunk index). CAR’s MRR point estimate exceeds the query-only hybrid at every α from 0.1 to 0.9 (0.422–0.440 vs. 0.344–0.395), so the aggregate trend is not an artifact of a single operating point; the aggregate margin is nonetheless not significant after Holm correction (Section 5.8).

At high overlap BM25 remains the best retriever (0.668), and on the lexical cell-value benchmark CAR stays within 0.03 of BM25 (0.573 vs. 0.599). An α-sweep shows CAR’s point estimate exceeds the hybrid’s at *every* fusion weight (Figure 10), so the aggregate trend is not an artifact of one operating point. The reading is not “CAR wins everywhere” but “CAR wins where the bias concentrates”: it is a *targeted* remedy for the low-overlap criterion queries that the diagnosis identifies, while the hybrid remains the pragmatic default elsewhere, a division of labor that would motivate routing queries to CAR only when low overlap is predicted. We describe that routing as a design implication rather than a validated policy, because two things it needs are missing. First, lexical overlap as measured here is computed against the gold chunk and is thus an evaluation-time quantity that a deployed system does not have: a deployed router would have to predict it from query-time signals (for example, the query’s content-morpheme overlap with its top BM25 candidates), and building and validating such a predictor is future work (Section 6.6). Second, at the lowest overlap the hypothesis-augmented dense channel alone posts a higher point estimate than the full fusion (0.333 vs. 0.271; Table 10), so the fusion’s value in that regime lies in retaining the lexical channel’s guarantees rather than in maximizing the point estimate, and a finer-grained router that also re-weights the channels per query is likewise left to future work. Finally, two disclosures. Throughout the paper, “CAR” denotes the fusion of (query + hypothesis) dense with criterion-expanded BM25; three sibling fusion configurations were evaluated alongside it and are disclosed in the Supplementary Material (Table S4) and the released artifacts, the best differing by ≤0.003 overall, with no configuration altering the significance pattern above. At the bin level the ranking of the siblings does vary, and we state the relevant case here rather than only in the limitations: on the low-overlap stratum the lexical-free sibling reaches 0.284 against the reported 0.271, a difference of 0.013 on 21 items that does not change which methods separate under Holm correction. We report the fusion that matches the architecture described above rather than the per-bin maximum and treat the selection itself as a source of multiplicity (Section 6.5). Also, the criterion-hypothesis prompt shares no examples with the QA-generation prompts (its few-shot examples come from unrelated domains; Supplementary Material S2), so the benchmark’s construction does not leak into the evaluated method. To our knowledge, CAR is the first retriever designed to target criterion containment rather than topical aboutness; whether that targeting separates it from *generic* hypothesis augmentation is examined below, and the margin, while consistent, does not yet reach significance.

To isolate the contribution of the *criterion* targeting itself (versus generic query augmentation), we substituted CAR’s criterion hypothesis with a generic HyDE [11] hypothesis in the identical fuse pipeline (Table 10, “Generic-HyDE fusion”). Criterion targeting wins consistently in point estimate (overall 0.425 vs. 0.389, and 0.271 vs. 0.213 in the bias-critical low-overlap regime; improving 37 items and worsening 23), but the paired difference is marginal rather than significant (ΔMRR +0.036, one-sided Wilcoxon *p* = 0.060, a single planned contrast outside the Holm family), and in repeated re-embedding runs the *p*-value moved between 0.04 and 0.06, a borderline effect we do not over-claim. Beyond the point estimates, criterion targeting also has structural advantages a generic hypothesis lacks: it produces short, register-constrained output in the vocabulary the corpus actually uses, is directly auditable against the diagnosed failure mode, and concentrates its effect exactly where the diagnosis predicts, an alignment a free-form paraphrase cannot claim. CAR is therefore best read as a *criterion-targeted* instance of query-side augmentation with a consistent but not-yet-confirmed statistical edge over generic hypothesis generation; a larger benchmark should settle it.

### 5.9. Efficiency and Cost

Because CAR adds a query-time language-model call, we quantify its cost (Table 11; per-query latency averaged over the benchmark; latencies were measured on an earlier 9179-chunk build of the retrieval-experiment index, and the 10% larger final index changes them negligibly). The following three index-only retrievers are all sub-100 ms per query: BM25 24 ms (local), dense 36 ms (dominated by the query-embedding API call), and the hybrid 64 ms. CAR is far heavier at ≈3.7 s per query, almost entirely the criterion-hypothesis LLM call (3.6 s; ≈318 input/93 output tokens, ≈$0.0024 per query); the remaining retrieval is identical to the hybrid. One-time corpus indexing (embedding 9179 chunks) costs ≈$0.34. CAR therefore trades two orders of magnitude of latency and a fraction of a cent per query for its low-overlap accuracy gain. This is acceptable for the offline or human-in-the-loop standards lookups we target, and reducible in latency-sensitive settings by caching hypotheses for recurring criterion queries or by delegating hypothesis generation to a small local language model (an 8B-class SLM), cutting latency and per-query cost by an order of magnitude and removing the external-API dependency. The accounting matters: CAR’s gain is not free, and where sub-100 ms latency is required the weighted-RRF hybrid remains the pragmatic default. The targeted-remedy profile of Section 5.8 suggests the natural deployment as follows: route a query to CAR only when low lexical overlap is predicted from query-time signals, paying the LLM latency exactly on the minority of queries where CAR’s advantage is significant.

### 5.10. Worked Examples and the Sensor-Criterion Stratum

The aggregate effects above (Section 5.2 and Section 5.4) have a concrete per-case form. Table 12 shows four real sections drawn from the counterfactual population, one each from the survey, bridge, expansion-joint, and tunnel domains. In every case a criterion-seeking query should retrieve the section that states the criterion in its body, but the plan-framed title buries it in full-corpus dense retrieval. Moving the body criterion into the title (the cf counterfactual) lifts the same document toward the top. Both ranks are measured in the same 10,144-chunk dense space, so the columns are directly comparable.

The pattern matters most where sensing systems depend on it. The 2018 bridge-inspection-facility section (4-4) specifies the structural criteria for the fixed gantries on which structural-health sensors are mounted; under its plan title (“개선방안 검토,” a review of an improvement plan) the criterion sits at rank 72, and surfacing it moves the section to rank 27. The 2009 expansion-joint section (5-15), which governs the joints that displacement and strain sensors monitor, moves from rank 41 to rank 2. The 2021 drone-LiDAR integrated-survey section (1–2) moves from rank 17 to rank 2. In each case the criterion is present in the body throughout; only its placement changes, along with the title framing the retriever keys on. These cases are the individual face of the +0.030 mean title effect (Section 5.2) and the rank recovery under reframing (Section 5.4). They also show why a criterion query issued against a plan-skewed corpus needs either a lexical channel or Criterion-Aware Retrieval to surface the governing standard.

Because our application setting is sensor-instrumented road infrastructure, we further isolate the questions whose criterion a sensing or monitoring system produces or consumes: 11 of the 50 cell-value questions and 15 of the 81 non-lexical questions (26 in total), spanning LiDAR and drone survey accuracy, fixed bridge-inspection and structural-health-monitoring facilities, wrong-way-driving detection, tunnel tele-monitoring and ventilation, and electronic tolling. Table 13 reports retrieval on this stratum. The framing-bias signature is present here too: dense retrieval is the weakest retriever on the sensor questions (cell-value MRR 0.292 against BM25’s 0.561, and 0.268 on the combined stratum, the lowest of the four methods), so the bias is not an artifact of non-sensor content. The prescription also reproduces in miniature: on the non-lexical sensor questions the lexical channel collapses (BM25 0.196) and dense (0.250), the hybrid (0.255), and CAR (0.219) all overtake it—the corpus-wide low-overlap crossover—while the hybrid attains the stratum’s best Recall@10 (0.846 combined), consistent with its role as the dependable default. These strata are small (*n* = 11, 15, 26) and we report them as descriptive, but they make the sensing relevance evidential rather than rhetorical: the bias we diagnose and the remedies we characterize are both measured on the sensor-criterion subset.

## 6. Discussion

### 6.1. Interpreting the Framing Bias: Aboutness over Containment

Our central finding is that dense retrievers over-weight what a passage is *about*, as signaled by its title, relative to the criterial content it actually *contains*. Rewriting only a section title from plan- to criterion-framing, with the body held fixed, raised similarity to a criterion query in 78 of 80 cases (Section 5.2). The query targets content that already lives in the unchanged body, so a retriever sensitive to *containment* should be indifferent to this edit. Dense retrieval is not.

The controls isolate why. With the plan title kept and the same criterion vocabulary added to the body—at its end (cfb) or even as its first line (cfs)—the criterion-titled variant still wins both contrasts (Table 3). The total title effect (+0.030) thus splits the following three ways: term frequency (+0.013), early body position (+0.009; the primacy bias of dense encoders [6,7], reproduced here), and a title-framing residual (+0.008, *d_z_* = 0.53) that survives when both are controlled. Neither presence nor early placement suffices. The fifth-variant control rules out one explanation of the remainder—adding the criterion to a title that keeps its plan framing gains nothing over the first body line (cfp ≈ cfs, n.s.), so the residual is not a matter of the title slot as such—and the sixth-variant control then divides it. Half is title token composition: deleting the plan tokens shortens the heading and moves it toward the query, and that alone contributes a significant share on both embedding families. The other half is what the title *asserts*, a plan rather than a criterion, which is significant on text-embedding-3-large (cfn−cfp, dz = 0.46) but not on embed-multilingual-v3.0 (Section 5.2). We therefore hold the aboutness reading for the total title effect, which is robust and cross-model, and treat its plan-semantics component as supported on one encoder rather than established in general. This is an aboutness effect in the sense of Hjørland [8], with the title operationalizing topicality, reproduced here in neural retrieval over Korean administrative text. The effect is not specific to OpenAI: an independent model family, Cohere embed-multilingual-v3.0, reproduces it (cf vs. orig Δ = 0.0206, 98%, *p* = 9.3 × 10^−15^, and *d_z_* = 1.64).

### 6.2. Relation to Literal Bias and a Pre-Empted Counterargument

This bias has a definite *direction* that distinguishes it from the “literal” biases reported by Fayyaz et al. [9], where dense retrievers favor short, early, and surface-literal matches. Our query and the criterial body share their lexical content; the title edit adds no new query terms. What moves the score is *where* the criterion concept is asserted, not whether its surface form is present. The two findings are complementary as follows: literal bias concerns surface matching, while framing bias concerns the framing content the title asserts.

The vocabulary-mismatch objection (Section 2.3) is closed by the same controls as follows: cfb and cfs inject the criterion into the body at matched frequency and dense retrieval still prefers the title placement, so neither lexical absence nor early position accounts for what remains (Section 5.2).

### 6.3. Prescriptive Implications

The retrieval results make the practical consequence concrete. On the cell-value benchmark, dense retrieval alone underperforms BM25 on every metric, and a weighted-RRF hybrid with a Korean morphological tokenizer recovers significantly over dense (Section 5.3). The lexical advantage is conditional, not intrinsic: stratified by query–document overlap (Section 5.7), BM25’s lead reverses below ≈0.5 overlap, and at ≤0.35 BM25 buries the target at a median rank of 166. The criterion queries most exposed to framing bias are exactly these low-overlap queries, and neither channel is strong there alone, so keeping both channels is not optional. A cross-encoder reranker recovers ranking quality (MRR@10 0.748, matching BM25) but not recall (0.892 vs. 0.962): it reorders the candidate pool and cannot retrieve criterion documents buried beyond it. The conclusion is narrow but firm. Dense retrieval deployed alone is risky for criterion queries over framing-skewed corpora, and reranking mitigates poor ordering but not the recall loss; a lexical channel must stay in the loop.

Index-side criterial reframing (Section 5.4) attacks the source instead, re-surfacing a body criterion in the indexed header. Under oracle extraction it nearly eliminates the burial (mean rank 254.8 → 4.6), which bounds what the lever could achieve—but no query-independent extractor transfers (all n.s.), because an index cannot anticipate which criterion a query will seek. Each stage thus leaves a residual: reranking cannot recover documents outside the pool, and index-time rewriting is structurally query-blind. What dependably helps is the lexical channel, which matches the body criterion to the query directly; this is why a hybrid is the pragmatic default. **Criterion-Aware Retrieval (CAR)** takes the remaining step at query time (Section 5.8), fusing a criterion-conditioned dense ranking with a criterion-expanded lexical ranking. Its profile is that of a *targeted* remedy: in the low-overlap regime where the bias concentrates, it beats both BM25 and the hybrid after Holm correction, while over the full set it significantly exceeds only dense. It does not, however, rank better than a cross-encoder reranker, which is both cheaper and faster (Section 5.8). What separates the two is the axis on which they act. Reranking reorders a fixed pool and inherits its recall exactly; CAR enlarges the pool. Since the failure this paper diagnoses is burial—criterion documents pushed outside the candidate set, where no reordering can reach them—the recall axis is the one the diagnosis implicates, and it is the one on which CAR leads (0.857 vs. 0.714 at low overlap). The practical division of labor follows, and it is a layered one rather than a choice: keep the hybrid as the default channel, add reranking for ordering, and widen the pool with CAR where missing the governing clause is the costly error. Routing CAR in on predicted-low-overlap queries remains the way to bound its latency cost, though the query-time overlap predictor itself has still to be built (Section 5.8).

### 6.4. Sensor-Relevance: From Sensed Parameters to Retrievable Standards

These design-specification documents are the authoritative reference layer for the sensing and monitoring systems deployed across the expressway network—vibration-based monitoring [25], deep-learning structural health monitoring [26,27], vision-based SHM [28], and wireless smart-sensor networks [29]—and they encode, as binding criteria, the parameters those systems both produce and consume [30]. Concrete examples recur throughout the corpus. Design speeds and traffic volumes from vehicle-detection-system (VDS) field surveys set geometric criteria. The section “고속도로 역주행 사고 예방 대책” (countermeasures for wrong-way driving) governs the installation of loop-, radar-, and vision-based wrong-way detectors. “드론라이다를 활용한 통합측량” specifies LiDAR survey-and-mapping accuracy standards, and its error table is the source of several of our QA items. The motivating section “교량 고정식 점검시설” specifies the fixed bridge-inspection facilities on which structural-health sensors are mounted. Tunnel water-quality tele-monitoring (TMS) sections set discharge thresholds that decide whether an automated monitoring station is installed at all. The dependency extends to emerging facility classes as follows: cable-supported pedestrian bridges under vibration monitoring, whose deflection and vibration criteria are still being consolidated, must draw their governing clauses from dispersed guideline corpora of exactly this kind—and suspension- and pedestrian-bridge provisions indeed recur in this corpus’s structures chapters. The same holds for the analysis and mitigation methods that such criteria govern, from transient response computation for shell structures [31] to low-frequency vibration and noise control with graded metamaterials [32]; we note these as instances of the engineering practice that consumes design criteria, not as retrieval methodology. In each case the binding criterion is the last mile of a sensor data lifecycle: measured in the field, codified into a specification, and later retrieved to inform a downstream decision.

This is where framing bias becomes a decision-integrity risk. A query such as “installation criteria for fixed bridge inspection facilities” must return the clause that states the criterion. If a dense retriever instead surfaces a plan-framed section—and in unedited full-corpus retrieval we observe the criterion-bearing targets of the framing-prone population buried at a mean rank near 255 (Section 5.4)—a field engineer or a digital-twin maintenance module may act on an aspirational improvement plan rather than the governing standard, under- or over-specifying the instrumentation or applying the wrong maintenance threshold. The retrieval layer we study is therefore not incidental to sensing; it determines whether sensor-informed engineering knowledge stays correctly actionable. On the 26 sensor-criterion questions isolated in Section 5.10 (Table 13), dense retrieval is the weakest retriever and the hybrid the most dependable (best Recall@10), so this is a measured risk rather than only an argued one. Stated plainly, for sensor-relevant queries what this study delivers is a diagnosed and measured risk plus a dependable default (the hybrid), not a demonstrated CAR gain on the sensor stratum itself, where CAR trails the hybrid (Table 13). Our results show that the layer can be hardened, since reframing under oracle extraction restores criterion-bearing specifications to the top of the ranking (Section 5.4), but also that doing so reliably in deployment is not yet solved. Retrieval integrity for sensor-informed standards is an open engineering problem, not a settled one.

### 6.5. Limitations and Threats to Validity

Several limitations bound our claims. First, although we expanded the Level 2 QA benchmark to *n* = 50 plus 81 non-lexical items, both remain modest, the low-overlap stratum on which CAR’s Holm-significant advantage rests has *n* = 21, and the cell-value answers of the lexical set favor BM25; we plan further expansion, broader category balance, and human relevance labeling. The layered validation of the non-lexical set (LLM adversarial review, deterministic grounding checks, and author review of every machine-flagged item) surfaced and corrected genuinely ungrounded premises in two of 81 items (2.5%), which bounds the set’s residual error rate but does not substitute for full-set human verification. A further threat is circularity: the same model family (Claude Sonnet 4.6) generates the QA items, adversarially validates them, and produces CAR’s criterion hypotheses. The deterministic grounding checks, the author’s review of every flagged item, and the disjoint-prompt control of Section 5.8 mitigate this but do not eliminate it. As a partial probe we regenerated the low-overlap stratum with an independent generator (GPT-4.1), holding the prompt, the screen and the index fixed; the eligible source pool is exhausted at 53 sections and nine items survived screening, on which the ordering reproduces (CAR 0.143 against 0.038 for the hybrid, 0.033 for dense and 0.018 for BM25; Table S8). At *n* = 9 no contrast approaches significance and we report the direction only, but it indicates the ordering is not an artifact of single-generator authorship. The following two further observations bound the concern rather than removing it: the deterministic grounding check alone rejected 51% of the GPT-4.1 candidates (27 of 53), whereas in the original pipeline the *preceding* LLM-adversarial pass rejected 35% (43 of 124) before that check ran (Section 5.7)—the screening therefore does substantive work on an independent generator’s output rather than admitting it wholesale; a benchmark large enough to replace the present one, with full human verification, remains future work. We also did not evaluate cross-encoder reranking applied to CAR’s widened pool; the division of labor described in Section 6.3 predicts the combination is complementary, but it is a prediction—measuring it is the natural next test and remains future work. Relatedly, four CAR fusion configurations were evaluated and the reported one selected as the architecture described in Section 5.8; the best-performing sibling differs by ≤0.003 overall and all four are disclosed in the Supplementary Material (Table S4), but their ranking varies at the bin level—on the low-overlap stratum the lexical-free sibling posts 0.284 against the reported 0.271—so the selection is a source of multiplicity we make explicit. We also disclose a sensitivity of the headline low-overlap result to benchmark repair: before the index-key repair described in Section 4.1 restored seven shadowed gold chunks, the low-overlap CAR-over-hybrid contrast did not survive Holm correction (*p*_Holm = 0.12 on the pre-repair set of 22 items, versus 0.035 after repair on 21); the repair corrected a measurement defect rather than selecting for significance, and we report the trajectory for completeness.

Second, the semantic component of the title residual is encoder-dependent as follows: significant on text-embedding-3-large and absent on embed-multilingual-v3.0 (Section 5.2). Two embedding families are too few to tell whether this reflects a property of administrative framing that some encoders are sensitive to, or a limitation of the shorter-context model; the token-composition component and the total title effect, which are what the prescriptive part of this paper rests on, replicate on both. Third, our bias evidence pairs a controlled counterfactual (causal; Section 5.2) with an observational prevalence study (Section 5.5). The latter is confounded by topic, since plan- and standard-framed groups differ in content as well as framing, and it operationalizes relevance as retrieval of the source document rather than human judgment, so human relevance labels and inter-annotator agreement remain future work. The counterfactual’s queries are, conversely, template-constructed (*topic* + criterion term): a deliberately controlled form that supports causal isolation but does not sample the phrasing variability of real user queries, which the independent QA benchmarks of Section 5.3 and Section 5.7 supply instead. Fourth, results come from a single corpus of Korean expressway design documents, so cross-domain generalization is future work.

Fifth, the parsing-pathway comparison (C3) is partial: HWPX and PDF are measured corpus-wide (Section 5.6), but the HML arm rests on the single HML export currently available and the HTML coordinate pathway is not yet measured, both of which require Hangul re-export to reach corpus scale and remain future work. The PDF pathway uses a PyMuPDF and pdfplumber union rather than alternative extractors such as Camelot or Docling, and our one-to-one table alignment scores cross-page table splits or merges as partial loss rather than re-stitching them; these are conservative-to-lenient choices, and we state them explicitly. Our significance tests, implemented in NumPy, were cross-validated against SciPy with matching *p*-values and effect sizes, so statistical implementation is not a residual concern.

### 6.6. Future Work

The immediate priorities are human relevance labels with inter-annotator agreement (weighted κ) to replace our source-document relevance proxy; a larger, more balanced QA benchmark with non-lexical answers (the current set favors BM25); and an end-to-end criterion extractor with measured precision/recall to remove the oracle assumption in reframing and verify end-to-end QA gains. We will also broaden the cross-model evidence beyond two embedding families, add cross-encoder and learned-sparse baselines, build and validate a query-time predictor of lexical overlap to operationalize the CAR routing of Section 5.8, and explore hybrid weighting that sets α dynamically per query, in the spirit of DAT [19], so that criterion queries receive more lexical weight while genuinely topical queries retain dense semantics.

## 7. Conclusions

We presented an end-to-end study of retrieval over Korean government technical documents, using two decades (2005–2024) of Korea Expressway Corporation design-practice guidelines as a corpus. This paper makes three contributions.

First, as an insight (C1), we identified and causally isolated a *framing bias* in dense retrieval over Korean administrative text as follows: embedding models over-weight the *aboutness* signaled by a section title over the *criterial containment* present in the body. A controlled counterfactual (*n* = 80) that holds the body fixed and rewrites only the title raises similarity in 78 of 80 cases with a large effect size and reproduces on a second embedding family (Table 3). The effect decomposes into term frequency, early body position—the known primacy bias, reproduced here—and a title residual that survives both. Two further controls anatomize that residual rather than assign it to one cause: it is not a title-slot effect, and a length-matched neutral-token substitution splits it into a token-composition component that replicates on both encoders and a plan-semantics component significant on one and absent on the other (Section 5.2). The framing account is thus established for the total title effect and qualified for its semantic component.

Second, as a remedy analysis (C2), we examined corrections at every stage and found each only partial. A retrieval-side hybrid improves significantly over dense but not over BM25 on lexical QA. A cross-encoder reranker recovers ranking quality but not recall, because it cannot retrieve documents buried beyond its candidate pool. Index-side *Criterial Reframing* establishes the mechanism’s ceiling under oracle extraction and harms no case, yet no query-independent extractor transfers, an index being structurally unable to anticipate which criterion a query will seek. Simple index-side manipulations of the title—removing it, weighting it separately, fusing title and body scores—are all worse than leaving the chunk intact, which is itself informative: the title is mis-weighted signal in one regime, not noise to be removed (Section 5.4). We propose Criterion-Aware Retrieval (CAR), which hypothesizes the sought criterion at query time and, in the low-overlap regime the bias creates, beats both BM25 and the hybrid after Holm correction. Measured against the strongest standard alternative, however, CAR does not rank best: a cross-encoder reranker ranks better at low overlap without a language-model call. What reranking cannot do is retrieve what its input pool omits—its recall is that of the channel it reorders—and omission is precisely the failure that framing bias produces, which is where CAR leads (Table 9). We therefore present CAR as complementary to reranking rather than superior to it and recommend a layered deployment: hybrid retrieval by default, reranking for ordering, and CAR to widen the pool where missing the governing clause is the costly error.

Third, as supporting engineering (C3), HWPX cell-coordinate parsing (26,757 tables) enabled both ground-truth-based evaluation and a Level 1 table-preservation comparison across parsing pathways. Structured formats are near-lossless (HML 97.3% of large-table cell content on a single-document probe), whereas PDF recovers 91.7% of large-table cell content, detects under half of all tables as tables, and varies from 75% to 98% across chapters. Pathway choice, not just format availability, governs how much tabular criterion content survives ingestion.

These findings matter for infrastructure decision-making. Design-criterion retrieval forms the knowledge backbone for bridge and road inspection, maintenance planning, and digital-twin reasoning, where a retriever that surfaces “plans” over binding “criteria” can misinform sensor placement and maintenance actions. On the 26-question sensor-criterion stratum this risk is measured rather than argued, though the stratum is small enough that we report it descriptively: dense retrieval is the weakest system there and the hybrid the most dependable (best Recall@10, 0.846; Section 5.10). What holds on exactly the queries a sensing system would ask is therefore the diagnosis and the default prescription—the hybrid—and not a demonstrated advantage for the method we propose, which trails the hybrid on that stratum.

We acknowledge the following main limitations: a modest QA set, a controlled probe paired with a topic-confounded observational study, and a single corpus. Future work will complete the four-format parsing comparison (corpus-scale HML and the HTML coordinate pathway, both pending Hangul re-export), expand the observational bias sample, add human relevance labels, and broaden the embedding-model evidence.

## Figures and Tables

**Table 1:** The three indices used in this study, and the dimensions on which they differ. Ranks and rank-based metrics are comparable only within a row.

Index	Span	Size	Chunk Composition	Store/Embedding Provenance	Used In
**Research corpus**	2005–2024	8041 chunks/1388 sections	guidelines only	local; embedded for this study	Section 5.1 and Section 5.2 (population, counterfactual)
**Operational index (Pinecone)**	2001–2024	9434 vectors/24 namespaces	guidelines, production chunking	Pinecone; production-time vectors	Section 5.3, Section 5.4 and Section 5.5 (Level 2, reframing oracle, prevalence)
**Retrieval-experiment index (local)**	2005–2024 + commentary	10,144 chunks	guidelines + commentary, keys made unique per source file	local; re-embedded at 1536 d	Section 5.2 (rank displacement), Section 5.7, Section 5.8, Section 5.9 and Section 5.10 (overlap strata, CAR, examples)

**Table 2:** Counterfactual variants. All share the identical original body text; cfb and cfs add one criterion mention (matching cf in term frequency) at the body’s end and start, respectively; cfp keeps the plan title intact and appends the criterion term to it (frequency-matched, title slot).

Variant	Title Framing	Body
**orig**	plan title	criterion content (original)
**cf**	criterion title (plan tokens removed)	criterion content (original)
**cfp**	plan title **+ criterion appended**	criterion content (original)
**cfn**	plan tokens **replaced by neutral tokens** of equal length, + criterion appended	criterion content (original)
**cfb**	plan title	+ one criterion mention at the body **end** (frequency-matched)
**cfs**	plan title	+ one criterion mention as the **first body line** (frequency- and position-matched)

**Table 3:** Controlled counterfactual results for the framing-bias probe (*n* = 80 sections, 2005–2024). Δ is the mean change in cosine similarity between the query and the document variant; “Cases” is the fraction of sections moving in the predicted direction. The OpenAI rows other than the cfn contrasts come from a single five-variant embedding batch with text-embedding-3-large; the two cfn contrasts come from a separate batch that reproduces the shared contrasts within 0.001 (Section S7). The three cross-model rows use Cohere embed-multilingual-v3.0. All *p*-values are one-sided, reflecting the directional hypotheses of Section 4.5.

Contrast	What It Isolates	Δ Cosine	Cases	Wilcoxon *p*	Cohen’s *d_z_*
**cf vs. orig (main)**	Total title effect	+0.0303	78/80 (98%)	1.1 × 10^−14^	1.63
**cfb vs. orig**	Term frequency (+1 body mention)	+0.0131	76/80 (95%)	2.4 × 10^−14^	1.18
**cfs vs. cfb**	Early body position (primacy)	+0.0091	65/80 (81%)	5.7 × 10^−9^	0.77
**cf vs. cfs**	**Title framing beyond position (aboutness)**	**+0.0081**	**59/80 (74%)**	**2.2 × 10^−5^**	**0.53**
**cf vs. cfb**	Position + slot combined	+0.0172	65/80 (81%)	6.7 × 10^−10^	0.90
**cf vs. orig (cross-model, Cohere)**	Total title effect, independent model	+0.0206	78/80 (98%)	9.3 × 10^−15^	1.64
**cfn vs. cfp**	**Plan semantics at matched length**	**+0.0048**	**53/80 (66%)**	**1.3 × 10^−4^**	**0.46**
**cf vs. cfn**	Title token composition (shortening)	+0.0040	54/80 (68%)	8.8 × 10^−5^	0.46
**cfn vs. cfp (cross-model, Cohere)**	Plan semantics, independent model	−0.0005	38/80 (48%)	0.50 (n.s.)	−0.09
**cf vs. cfn (cross-model, Cohere)**	Token composition, independent model	+0.0045	66/80 (83%)	1.1 × 10^−8^	0.76

**Table 4:** Level 2 retrieval results on the 50-item QA benchmark with the pure harness (operational index, Section 4.1). Best value per column in bold. “Dense + cross-encoder” reranks dense’s top 50 candidates with Cohere rerank-multilingual-v3.0.

System	Recall@10	MRR@10	nDCG@10
**BM25 (Kiwi)**	**0.962**	0.740	**0.777**
**Dense (text-embedding-3-large)**	0.757	0.417	0.476
**Dense + cross-encoder rerank**	0.892	**0.748**	0.762
**Hybrid (weighted RRF, α = 0.3)**	0.923	0.526	0.625
**Hybrid (weighted RRF, α = 0.5)**	0.854	0.546	0.613

**Table 5:** Criterial Reframing on full-corpus retrieval (*n* = 78 framing-prone cases; 9434-vector corpus). Reframing the indexed header restores buried target documents; reframed values in bold.

Metric	Baseline (Plan Title)	Reframed
**MRR**	0.418	**0.686**
**Mean rank**	254.8	**4.6**
**Median rank**	4	**1**
**Recall@1**	28.2%	**55.1%**
**Recall@5**	61.5%	**84.6%**
**Recall@10**	71.8%	**88.5%**
**Improved/worsened (of 78)**	—	**51/0**

**Table 6:** Observational prevalence in unedited full-corpus dense retrieval (operational index, Section 4.1): plan-framed vs. standard-framed criterion-bearing sections. Uncorrected Fisher’s exact tests (one-sided): >rank 10, *p* = 0.015, OR = 2.6; >rank 50, *p* = 0.042, OR = 3.7. After Holm correction across the three correlated tests, only the >rank-10 burial-rate difference remains significant (*p*_Holm = 0.045); the >rank-50 contrast (*p*_Holm = 0.084) and the full-distribution test (Mann–Whitney *p* = 0.058) are exploratory.

Group	*n*	Mean Rank	Median	>Rank 10	>Rank 50
**Plan-framed (*bangan*)**	78	141.2	4	27%	12%
**Standard-framed (*gijun*)**	88	121.8	3	12%	3%

**Table 7:** Level 1 table preservation against HWPX cell-coordinate ground truth (2024 corpus). *Cell preservation* is the share of ground-truth cell values recovered; *detection* is the share of ground-truth tables with ≥50% of cells recovered. HWPX is the ground-truth source (100% reference, also serving as a harness self-test); HML is a single-document probe; PDF spans all eight chapters. “≥2 × 2” restricts to tables of at least two rows and two columns.

Format	Scope	Docs	GT Tables	GT Cells	Cell Preservation	Table Detection
**HWPX (source = GT)**	≥2 × 2	8	390	7393	**100.0%**	100.0%
**HML (1-doc probe)**	≥2 × 2	1	74	1129	**97.3%**	100.0%
**PDF (union)**	≥2 × 2	8	390	7393	**91.7%**	87.7%
**PDF (union)**	all	8	1069	8537	81.5%	**47.6%**

**Table 8:** Gold-chunk retrieval on non-lexical QA, stratified by query–document lexical overlap (10,144-chunk index; BM25 with Kiwi tokenizer vs. dense text-embedding-3-large). Lower overlap means the question shares fewer surface terms with its answer. Best MRR per row in bold.

Query–Answer Lexical Overlap	*n*	BM25 MRR	Dense MRR	BM25 Recall@10	Dense Recall@10
**Lexical QA (Section 5.3 set, re-run on this index)**	50	**0.599**	0.420	1.000	0.840
**Non-lexical, all**	81	**0.343**	0.289	0.481	0.593
**Non-lexical, high (>0.50)**	33	**0.668**	0.375	0.848	0.697
**Non-lexical, mid (0.35–0.50)**	27	0.176	**0.231**	0.296	0.556
**Non-lexical, low (≤0.35)**	21	0.048	**0.230**	0.143	0.476

**Table 9:** Criterion-Aware Retrieval (CAR) vs. baselines on non-lexical QA, by query–document lexical-overlap bin (10,144-chunk index; MRR/Recall@50; best MRR and best Recall@50 per row in bold, ties unbolded; the lexical row reports MRR only). CAR = weighted-RRF of (query + criterion-hypothesis) dense and criterion-expanded BM25. The two reranking rows apply Cohere rerank-multilingual-v3.0 to the top 50 candidates of the respective channel; their Recall@50 is by construction that of the channel that feeds them. The dense channel of this run reproduces the archived Dense row to 0.001 on the non-lexical aggregate (≤0.004 at the bin level) and the CAR row exactly; the reconstructed hybrid channel differs from the archived hybrid by 0.011 MRR on the non-lexical aggregate (up to 0.031 in the high bin, a fusion-pool-depth effect), and we report the archived values in the dense and hybrid rows for continuity (Section S9).

Overlap Bin	*n*	BM25	Dense	Hybrid	Dense+Rerank	Hybrid+Rerank	CAR (Ours)
**Lexical QA (cell-value, re-run on this index)**	50	0.599	0.420	0.599	0.662	**0.674**	0.573
**Non-lexical, all**	81	0.343/0.741	0.289/0.815	0.395/0.889	**0.480**/0.815	0.458/0.839	0.425/**0.938**
**High (>0.50)**	33	**0.668**/1.000	0.375/0.909	0.610/1.000	0.585/0.909	0.585/0.939	0.563/1.000
**Mid (0.35–0.50)**	27	0.176/0.778	0.231/0.778	0.347/0.889	**0.432**/0.778	0.418/0.815	0.376/**0.926**
**Low (≤0.35)**	21	0.048/0.286	0.230/0.714	0.120/0.714	**0.376**/0.714	0.310/0.714	0.271/**0.857**

**Table 10:** CAR ablation: component contributions on non-lexical QA (MRR; 10,144-chunk index). The criterion *hypothesis* lifts the semantic channel across all bins; criterion *expansion* lifts the lexical channel at low-to-mid overlap; the fusion (CAR) is best overall and at mid overlap, while at the lowest overlap the hypothesis-augmented dense channel alone is strongest.

Component	All (81)	High (33)	Mid (27)	Low (≤0.35, 21)
**Dense (query only)**	0.289	0.375	0.231	0.230
**Dense + criterion hypothesis**	0.398	0.494	0.332	**0.333**
**BM25 (query only)**	0.343	**0.668**	0.176	0.048
**BM25 + criterion expansion**	0.419	0.666	0.340	0.135
**Generic-HyDE fusion (non-targeted)**	0.389	0.562	0.313	0.213
**CAR (criterion-targeted fusion)**	**0.425**	0.563	**0.376**	0.271

**Table 11:** Per-query retrieval latency and added cost (latencies averaged over the benchmark). Latencies were measured on an earlier 9179-chunk build of the retrieval-experiment index; the final index is 10% larger, and because dense search is a single matrix product and the LLM call dominates CAR’s latency by two orders of magnitude, the difference is below the reporting precision of this table. “Added cost” excludes the one-time corpus indexing (≈$0.34, 2.63 M tokens). All *p*-values reported in this paper are one-sided (Section 4.5).

Method	Latency/Query	Added Cost/Query	Dominant Cost
**BM25**	24 ms	—	local compute only
**Dense**	36 ms	<$10^−5^	query embedding
**Hybrid**	64 ms	<$10^−5^	dense + BM25 + RRF
**CAR (ours)**	≈3685 ms	≈$0.0024	criterion-hypothesis LLM call (3588 ms)

**Table 12:** Worked examples of framing bias in individual sections (real cases from the counterfactual population; ranks out of the 10,144-chunk index, measured in the same dense space). “Dense rank” is the target’s full-corpus rank under its original plan-framed title; “surfaced” is its rank when the body criterion is moved into the title (the cf counterfactual). Δcos is the corresponding change in query–document cosine similarity.

#	Section (Year)	Criterion the Query Seeks	Plan-Framed Title (Gloss)	Body Criterion	Dense Rank → Surfaced	Δcos
**1**	Drone-LiDAR integrated survey (2021, 1–2)	fee standard for drone integrated survey	“드론 통합측량 추진방안” (plan to adopt drone integrated survey)	대가기준 (fee standard)	17 → 2	+0.049
**2**	Bridge inspection facilities (2018, 4–4)	structural standard for the facilities	“교량 점검시설 …개선방안 검토” (review of an improvement plan)	구조기준 (structural standard)	72 → 27	+0.034
**3**	Expansion joints (2009, 5–15)	type standard for expansion joints	“신축이음장치 개선 적용방안” (improvement application plan)	형식기준 (type standard)	41 → 2	+0.092
**4**	Tunnel-exit glare-reduction facility (2012, 7–8)	orientation standard for the facility	“터널 출구부 직광 저감시설 설치방안” (installation plan)	방위기준 (orientation standard)	47 → 21	+0.019

**Table 13:** Retrieval on the sensor-criterion stratum (questions whose criterion a sensing or monitoring system produces or consumes; MRR, 10,144-chunk index, best per row in bold). Strata are small and reported as descriptive. Dense is the weakest retriever on the sensor questions; the hybrid is the most dependable (best combined MRR tie and best Recall@10, 0.846).

Sensor-Criterion Stratum	*n*	BM25	Dense	Hybrid	CAR
**Cell-value (lexical)**	11	**0.561**	0.292	0.480	0.480
**Non-lexical**	15	0.196	0.250	**0.255**	0.219
**Combined**	26	**0.350**	0.268	**0.350**	0.330

## Data Availability

The corpus (Korea Expressway Corporation design-practice guidelines, 2005–2024) is access-restricted and not publicly available owing to licensing and confidentiality constraints; it is analyzed and reported here with the permission of the Korea Expressway Corporation. Aggregated statistics and anonymized samples supporting the reported findings are included in the paper. The evaluation and analysis code, the counterfactual-probe harness, and the derived artifacts sufficient to reproduce every reported statistic (Section 4.7) will be released in a public repository with a DOI upon publication.

## Supplementary Materials

The following supporting information can be downloaded at https://www.mdpi.com/article/10.3390/s26175683/s1, Section S1: QA-generation prompts (non-lexical and low-overlap generators); Section S2: CAR criterion-hypothesis prompt (leakage-free) and generic-HyDE baseline prompt; Section S3: relevance-audit prompt; Section S4 (Table S4): CAR fusion-variant disclosure; Section S5: human review of machine-flagged benchmark items; Section S6 (Table S6): fifth-variant counterfactual control (cfp) and cross-model decomposition; Section S7 (Table S7): sixth-variant control (cfn), the plan-semantics decomposition, and the cross-model degeneracy disclosure; Section S8 (Table S8): independent-generator probe (gpt-4.1); Section S9: BM25 index reconstruction underlying the reranking baseline; Section S10 (Table S10): index-side title baselines.

## Abbreviations

The following abbreviations and notation are used in this manuscript:

**Term**	**Meaning**
RAG	retrieval-augmented generation; here, only its retrieval stage is studied
Dense retrieval	ranking by cosine similarity of text embeddings (text-embedding-3-large, 1536-d)
BM25	classical lexical (term-matching) ranking; Kiwi morphological tokenizer
RRF/α	reciprocal rank fusion (k = 60) merging dense and lexical rankings; α weights the two lists
Hybrid	weighted-RRF fusion of dense and BM25
CAR	Criterion-Aware Retrieval (ours): an LLM hypothesizes the sought criterion at query time
HyDE	hypothetical-document query expansion (generic baseline for CAR)
HWPX/HML	XML-based (and legacy-XML) formats of the Korean Hangul word processor
MRR@10	mean reciprocal rank truncated at 10: average of 1/rank of the gold chunk, 0 if below rank 10 (Table 4 and Section 5.3)
MRR	mean reciprocal rank without a cutoff: average of 1/rank over the full ranking (Section 5.4, Section 5.7, Section 5.8, Section 5.9 and Section 5.10; Table 8, Table 9, Table 10 and Table 13). See Section 4.5
Recall@10	fraction of questions whose gold chunk appears in the top 10
nDCG@10	rank-discounted relevance gain, normalized
Cohen’s *d_z_*	paired effect size: mean of per-item differences divided by their SD
Holm correction	family-wise multiple-comparison adjustment applied within each pre-specified test family
Lexical overlap	share of a question’s content morphemes that also occur in its gold chunk
GT	ground truth (cell-coordinate values extracted from the source HWPX XML)
SHM/VDS/TMS	structural health monitoring/vehicle detection system/tele-monitoring system
