# Supplementary material for: Reliable Criterion Retrieval for Sensor-Instrumented Road Infrastructure: Diagnosing and Correcting a Title-Framing Bias in Dense Retrieval over Korean Design Documents
Source: Sensors (Basel). 2026 Sep 7;26(17):5683. doi: 10.3390/s26175683 (PMC13568054; doi:10.3390/s26175683)
Supplement: Supplementary file 1 [file sensors-26-05683-s001.zip › sensors-4478214-supplementary.pdf]

Supplementary Material

Reliable Criterion Retrieval for Sensor-Instrumented Road Infrastructure: Diagnosing and Correcting a Title-Framing Bias in Dense Retrieval over Korean Design Documents

This supplement lists, verbatim, the language-model prompts referenced in Section 4.7 of the manuscript, together with the CAR fusion-variant disclosure (Section 5.8), the human review of machine-flagged benchmark items (Sections 5.7 and 6.5), the fifth- and sixth-variant counterfactual controls (Section 5.2), the independent-generator probe (Section 6.5), the index reconstruction underlying the reranking baseline (Section 5.8), and the index-side title baselines (Section 5.4). All prompts are in Korean, the language of the corpus; an English gloss follows each. Every language-model step uses `claude-sonnet-4-6` with default decoding, except the independent-generator probe of Section S8, which uses `gpt-4.1` by design.

S1. QA-Generation Prompts (Section 4.2 / Section 5.7)

S1.1 Non-lexical (paraphrase/inference) generator

```
너는 한국 도로설계 실무지침을 평가할 검색 벤치마크를 만드는 전문가다.
주어진 본문 단락 하나만으로 답할 수 있는 한국어 질문 1개를 만든다.
핵심 제약(반드시 지킬 것):
(1) 질문은 본문의 고유 명사·전문용어를 그대로 쓰지 말고 **동의어/일반화/풀어쓰기**로
바꾼다
(예: '핑거형 신축이음장치'→'교량 이음부 장치', '확충'→'늘리는').
(2) 답은 단순 수치나 본문에 통째로 있는 구절이 아니라, 이해가 필요한 **개념·조건·이유**가
되게 한다.
(3) 답은 반드시 이 단락으로 검증 가능해야 한다(환각 금지).
JSON 만 출력: {"question":..., "answer":..., "support_quote":<답 근거가 된 본문 구절
그대로>,
"category":"paraphrase|inference|multi_synth", "avoided_terms":[본문에서 일부러 피한
용어들]}
```

Gloss: generate one Korean question answerable from the given passage alone, paraphrasing away the passage's proper nouns and terms of art; the answer must be a concept/condition/reason verifiable from the passage; output JSON with question, answer, verbatim supporting quote, category, and avoided terms.

S1.2 Low-overlap (strict) generator

너는 한국 도로설계 실무지침 검색 벤치마크에서 *의미 검색만으로 풀 수 있는* 어려운 질문을 만든다.
주어진 본문 단락 하나로만 답할 수 있는 한국어 질문 1개를 만들되, **최대한 본문 어휘를 쓰지 않는다.**
엄격 제약:
(1) 질문에 본문의 고유명사·제품명·전문용어·약어·수치를 **단 하나도 그대로 쓰지 말 것.**
대상은 오직 '기능·역할·상황'으로만 일반적으로 묘사한다
(예: 'GFRP 보강근'→'금속을 대체하는 새 보강 재료', '소성변형'→'바퀴자국처럼 영구히 패는 현상',
'신축이음'→'온도변화로 늘었다 줄었다 하는 이음부').
(2) 질문 형태는 가급적 '왜', '어떤 조건에서', '무엇이 바뀌었고 그 이유는', '둘 중 어느 쪽이며 왜' 같은
개념·이유·조건·비교형으로 한다(단순 값 묻기 금지).
(3) 그럼에도 답은 반드시 이 단락으로 유일하게 검증 가능해야 한다(환각·복수정답 금지).
JSON 만 출력: {"question":..., "answer":..., "support_quote":<본문 근거 구절 그대로>, "category":"inference|multi_synth|comparison", "avoided_terms":[일부러 피한 본문 용어들]}

Gloss: as S1.1, but the question may not reuse a single proper noun, product name, term of art, abbreviation, or number from the passage; the target may only be described by function, role, or situation.

The multi-year expansion generator (Section 5.7, the `qa_nx*` items) reuses prompt S1.1 over the 2015–2024 corpus.

S2. CAR Criterion-Hypothesis Prompt (Section 5.8)

S2.1 Prompt used for all reported results (leakage-free)

너는 한국 기술 지침·설계 기준 문서 전문가다. 주어진 질의가 찾는 '기준'을 담은 짧은 가상의 지침 문장
(1~2 문장)을 작성한다. 일상어·풀어쓴 질의를 실제 지침에 쓰일 전문용어·기준 표현으로 바꿔라
(예: '수술실 공기를 얼마나 자주 갈아야 하는지'→'수술실 시간당 환기횟수 기준',
'엘리베이터가 층에 설 때 허용되는 어긋남'→'승강기 착상 정확도 기준').
정답을 모른다고 가정하고 질의 의도에 맞는 기준 진술만 생성한다. 문장만 출력(따옴표·머리말 없이).

Gloss: write a short hypothetical guideline sentence stating the criterion the query seeks, converting everyday phrasing into the technical criterion register; assume the answer is unknown.

Leakage control. The prompt's few-shot examples are drawn from domains unrelated to the corpus (hospital ventilation, elevator leveling). An earlier development version used road-domain examples that mirrored the QA generators' paraphrase examples in reverse; to rule out benchmark-construction leakage into the evaluated method, all reported results use the prompt above, re-generating every hypothesis. The headline numbers were essentially unchanged by this substitution (aggregate MRR differing by less than 0.002), confirming that any such leakage was not driving the result.

S2.2 Generic-HyDE baseline prompt (Table 9)

```
주어진 질의에 답하는 듯한 짧은 가상의 문단을 1~2 문장으로 작성하라.
정답을 모른다고 가정하고 자연스럽게 답을 추정해 서술한다. 문장만 출력.
```

Gloss: standard HyDE—write a short hypothetical passage that appears to answer the query, without criterion targeting.

S3. Relevance-Audit Prompt (Section 5.5)

```
너는 정보검색 평가의 relevance 심사관이다. 한국 도로설계 지침 검색에서, 주어진 [질의]가
찾는 '기준(criterion)'을 [문서]가 실제로 담고 있는지 판정한다.
relevant=문서가 그 기준을 직접 명시/규정함, partial=관련 주제이나 그 기준 자체는 약하게만
다룸,
not=그 기준을 담지 않음(주제만 겹치거나 무관).
JSON 만: {"relevance":"relevant|partial|not", "reason":"한 줄 근거"}
```

Gloss: judge whether the document actually states the criterion the query seeks (relevant/partial/not), with a one-line reason.

S4. CAR Fusion-Variant Disclosure (Section 5.8)

Four fusion configurations were evaluated on the identical index and benchmark; "CAR" in the manuscript denotes the architecture described in Section 5.8 (query+hypothesis dense ⊕ criterion-expanded BM25). Non-lexical MRR (untruncated) (n=81, the 10,144-chunk retrieval-experiment index of Section 4.1, final human-review-corrected benchmark).

Table S4. CAR fusion-variant disclosure: non-lexical MRR (untruncated) of the four evaluated configurations (n=81; low stratum ≤0.35, n=21). "CAR" is the configuration reported in the manuscript.

Configuration	Fusion	all (81)	low (≤0.35, 21)
CAR (plain)	hypothesis-dense ⊕ expanded-BM25	0.379	0.251
CAR+q (3-way)	query-dense ⊕ hypothesis-dense ⊕ expanded-BM25	0.426	0.261

**CARqh (reported as "CAR")**	(query+hypothesis)-dense ⊕ expanded-BM25	**0.425**	**0.271**
CARsem	query-dense ⊕ hypothesis-dense (no lexical)	0.354	0.284

The best sibling (CAR+q) differs from the reported configuration by 0.001 overall, and no configuration alters the significance pattern reported in Section 5.8. The selection of CARqh is architectural (it is the configuration described in the method) but constitutes a source of multiplicity, which Section 6.5 discloses.

S5. Human Review of Machine-Flagged Items (Sections 5.7 and 6.5)

All 81 retained non-lexical items passed a deterministic grounding check (verbatim quote and answer-value matching against the gold chunk); all 11 machine-flagged items were reviewed by the domain-expert author against the full source chunks. Nine proved grounded once the evidence excerpt was extended verbatim from the source; two (2.5%) carried genuinely ungrounded premises—one question presupposed a structure type absent from the source, one answer invented a meeting-round breakdown—and were corrected to the source's wording before final scoring. The review record (verdicts, rationales, and corrections) is included in the released artifacts.

S6. Fifth-Variant Control (cfp) and Cross-Model Decomposition (Section 5.2)

A single six-text embedding batch (query + `orig`/`cf`/`cfp`/`cfs`/`cfb` per section, n=80) was run per model: OpenAI `text-embedding-3-large` (1,536-d) and Cohere `embed-multilingual-v3.0` (asymmetric `search_query`/`search_document` embedding). `cfp` keeps the plan title intact and appends the criterion term to it (frequency-matched). Cohere contrasts involving `cfb` are omitted because its 512-token context can truncate a body-end addition. Within this batch every four-variant Δ of Table 3 reproduces within 0.001 (e.g., `cf−orig` +0.0304 vs. the reported +0.0303).

Table S6. Fifth-variant decomposition (paired Wilcoxon, Cohen's d_z; "cases" = share moving in the listed direction).

Contrast	Δ (OpenAI)	Cases	p	d_z	Δ (Cohere)	Cases	p	d_z
`cfp` vs. `cfs` (title slot, plan retained)	−0.0004	40/80	0.67 (n.s.)	−0.04	−0.0000	0/80	1.0 (n.s.)	−0.11
`cf` vs. `cfp` (plan-framing removal)	+0.0087	64/80	$1.1{\times}10^{-9}$	0.83	+0.0040	53/80	$2.7{\times}10^{-5}$	0.54
`cf` vs. `cfs` (Table 3 residual, this batch)	+0.0083	59/80	$2.0{\times}10^{-5}$	0.54	+0.0040	53/80	$2.7{\times}10^{-5}$	0.54
`cfp` vs. `orig` (criterion appended, plan kept)	+0.0217	75/80	$1.9{\times}10^{-14}$	1.38	+0.0166	79/80	$8.7{\times}10^{-15}$	1.66

`cf` vs. `orig` (total, reproduction)	+0.0304	78/80	1.0×10^{-14}	1.66	+0.0206	78/80	9.7×10^{-15}	1.63

Interpretation: with the plan framing retained, the title slot confers no advantage over the first body line (`cfp` vs. `cfs`, n.s. on OpenAI; the Cohere side of this contrast is uninformative: in this batch the two variants receive identical embeddings in 79 of 80 sections, and the one remaining case—differing by 5.7×10^{-4} in cosine—alone carries the printed d_z; in the batch of Section S7 the degeneracy is complete); the residual of Table 3 is carried by the removal of the plan framing, i.e., by the title's framing content.

S7. Sixth-Variant Control (cfn) and the Plan-Semantics Decomposition (Section 5.2)

The `cfp` control of Section S6 removes the plan framing and the plan tokens together, so the residual it isolates admits the following two readings: the *semantics* of plan vocabulary, or the mere *token composition* of the title. To separate them we added a sixth variant. `cfn` replaces the plan tokens of the title with neutral tokens **of equal length** and appends the criterion exactly as `cfp` does; `cfn` and `cfp` therefore differ only in whether the retained title tokens carry plan semantics, while `cf` and `cfn` differ only in title token composition.

The substitution dictionary was fixed before the run and applied by rule, with no per-case adjustment (eight entries, each a two-syllable Sino-Korean noun replaced by a two-syllable neutral noun): 방안→부문, 개선→영역, 검토→항목, 계획→측면, 조정→범위, 추진→부분, 제고→대상, 강화→요소.

A separate six-text batch (query + `orig`/`cf`/`cfn`/`cfp`/`cfs` per section, n=80) was embedded per model. As in Section S6, `cfb` is omitted from the cross-model batch because Cohere's 512-token context can truncate a body-end addition.

Table S7. Sixth-variant decomposition (paired Wilcoxon, Cohen's d_z; "cases" = share moving in the listed direction).

**Contrast**	**Δ (OpenAI)**	**Cases**	***p***	d_z	**Δ (Cohere)**	**Cases**	***p***	d_z
`cfn` vs. `cfp` (plan semantics, length matched)	+0.0048	53/80	1.3×10^{-4}	0.46	−0.0005	38/80	0.50 (n.s.)	−0.09
`cf` vs. `cfn` (title token composition)	+0.0040	54/80	8.8×10^{-5}	0.46	+0.0045	66/80	1.1×10^{-8}	0.76
`cf` vs. `cfp` (sum of the two rows above)	+0.0088	63/80	1.6×10^{-9}	0.82	+0.0040	54/80	2.3×10^{-5}	0.54
`cf` vs. `cfs` (Table 3 residual, this batch)	+0.0082	58/80	3.6×10^{-5}	0.53	+0.0040	54/80	2.3×10^{-5}	0.54
`cfp` vs. `cfs` (title slot, plan retained)	−0.0006	41/80	0.55 (n.s.)	−0.06	0.0000	0/80	1.00 (degenerate)	0.00

`cf` vs. `orig` (total title effect, reproduction)	+0.0306	78/80	1.0×10^{-14}	1.64	+0.0206	78/80	1.0×10^{-14}	1.63

On `text-embedding-3-large` the decomposition is additive to the reported precision: the plan-semantics component (+0.0048) and the token-composition component (+0.0040) sum exactly to the `cf−cfp` residual (+0.0088).

Degeneracy disclosure (Cohere). On `embed-multilingual-v3.0` the `cfp` and `cfs` variants receive *identical* embeddings for all 80 sections. The two variants differ only in whether a space or a newline separates the appended criterion from the title, and this encoder's preprocessing normalizes that difference; we verified that the underlying texts differ in all 80 cases. Cohere's `cf−cfp` and `cf−cfs` are therefore not two contrasts but one, which is why they agree exactly in the table above. It follows that the "`cfp` ≈ `cfs` on Cohere" observation reported in an earlier version of this analysis carries no information, and we do not rely on it; the corresponding OpenAI contrast, where the two texts do receive distinct embeddings, is unaffected.

Interpretation: the token-composition component replicates on both embedding families and is in fact larger on Cohere, whereas the plan-semantics component is significant on `text-embedding-3-large` and absent on `embed-multilingual-v3.0`. Section 5.2 states the resulting limit on what may be attributed to plan semantics alone.

S8. Independent-Generator Probe (Section 6.5)

Because the same model family (Claude Sonnet 4.6) generates the non-lexical QA items, adversarially validates them, and produces CAR's criterion hypotheses, the benchmark and the method share an author. As a partial probe we regenerated the low-overlap stratum with an independent generator, OpenAI `gpt-4.1`, holding the following three things fixed: the generation prompt (Section S1.2), the deterministic grounding screen, and the retrieval index.

Two properties of the regenerated set bound what it can show. The eligible source pool is exhausted at 53 sections, so the probe cannot be enlarged without changing the corpus. The deterministic grounding check alone rejected 51% of the `gpt-4.1` candidates (27 of 53), whereas in the original pipeline the *preceding* LLM-adversarial pass rejected 35% (43 of 124) before that check ran (Section 5.7)—the screening therefore does substantive work on an independent generator's output rather than admitting it wholesale. Nine items survived screening (mean query–gold lexical overlap 0.22, cap ≤0.35).

Table S8. Low-overlap stratum regenerated with `gpt-4.1` (n=9). Median gold rank is over 10,144 chunks.

System	MRR	Recall@10	Median gold rank
BM25	0.018	0.000	320
Dense	0.033	0.111	145

Hybrid	0.038	0.111	268
CAR (ours)	**0.143**	**0.222**	**100**

Paired one-sided Wilcoxon against CAR: BM25 p=0.19 (2 items improved, 0 worsened), Dense p=0.21 (2 improved, 1 worsened), Hybrid p=0.50 (1 improved, 1 worsened). At n=9 no contrast approaches significance, and we report the ordering only. The probe indicates that the ordering is not an artifact of single-generator authorship; it does not replace a benchmark large enough to test the claim independently, which remains future work.

S9. BM25 Index Reconstruction for the Reranking Baseline (Section 5.8)

The reranking rows of Table 9 require the BM25 channel on the non-lexical evaluation surface. The analysis machine's application-control policy blocks the native library of the Korean morphological analyser (`kiwipiepy`), so the archived BM25 index could not be rebuilt in place. We instead reused an index constructed for a separate study over the same corpus, after establishing that it occupies the identical key space—10,144 chunks in the same order—and that the tokenized forms of all 131 evaluation queries were cached.

Verification was run before any reranking call. The reconstructed index agrees with the archived run at the metric level to four decimal places in every overlap stratum (MRR, Recall@10, and Recall@50). Rank-exact agreement is 121 of 131 queries; all ten disagreements occur at rank 26 or below and arise from tie-breaking among chunks with equal BM25 scores, so none can affect MRR, Recall@10 or Recall@50. The acceptance gate was accordingly set at metric equality rather than rank identity, and the reranking experiment proceeded only after it passed. The verification output—per-query rank agreement and the tie diagnostics behind the 121-of-131 figure—is released as `bm25_reconstruction_check.json`.

S10. Index-Side Title Baselines (Section 5.4)

Five index-side configurations were evaluated with dense retrieval throughout (`text-embedding-3-large`, 1,536-d) on the same 10,144-chunk index and 131-query benchmark as Table 9. The title/body split rule was fixed before the run: the leading contiguous heading lines of a chunk constitute its title and the remainder its body; all 10,144 chunks resolve under the rule (0 missing titles, 0 empty bodies). The whole-chunk control reproduces the archived dense channel exactly on Recall@10 (0.687) and to 0.002 on MRR, validating the harness. The field-weighted arm mixes the two cosine similarities as $(1-w)\cdot$body + $w\cdot$title and was swept over $w \in \{0.0, 0.1, \ldots, 1.0\}$; w=0.2 is the best overall operating point and is the row shown, with the full grid in the released artifact (`title_baselines.json`). Late fusion applies reciprocal-rank fusion to the title-only and body-only rankings; max-pooling takes the maximum of the two similarities per chunk.

Table S10. Index-side title baselines: MRR by overlap stratum (131-query surface) with overall Recall@10; the final column is the 80-target framing-prone surface (construct caveat below). Best value per column in bold.

Configuration	Overall MRR	Overall R@10	Lexical (50)	High (33)	Mid (27)	Low (21)	Framing80
Whole chunk (control)	**0.341**	**0.687**	0.424	**0.379**	0.230	**0.229**	0.440
Body only (title removed)	0.243	0.504	0.292	0.231	0.237	0.152	0.376
Field-weighted (best overall *w*=0.2)	0.287	0.550	0.381	0.250	0.237	0.183	0.461
Late fusion (title ⊕ body)	0.230	0.466	0.386	0.163	0.145	0.072	0.431
Max-pooling	0.301	0.550	**0.436**	0.248	**0.246**	0.135	**0.604**

On the 131-query surface no configuration matches the control overall or on the low stratum; max-pooling, body-only and field-weighted edge the control on the mid stratum (0.246, 0.237 and 0.237 against 0.230) and max-pooling on the lexical stratum (0.436 against 0.424)—differences ≤0.016 to which we attach no claim. **Construct caveat (Framing80).** The 80-target surface uses queries derived from section titles (Section 4.4), so configurations that up-weight the title win by construction—the title-only endpoint (*w*=1.0) reaches 0.604, tied with max-pooling—and this surface cannot rank retrieval quality; it is reported for completeness.
